# Optogenetic engineered umbilical cord MSC-derived exosomes for remodeling of the immune microenvironment in diabetic wounds and the promotion of tissue repair

**DOI:** 10.1186/s12951-023-01886-3

**Published:** 2023-06-02

**Authors:** Xin Zhao, Luoqin Fu, Hai Zou, Yichen He, Yi Pan, Luyi Ye, Yilin Huang, Weijiao Fan, Jungang Zhang, Yingyu Ma, Jinyang Chen, Mingang Zhu, Chengwu Zhang, Yu Cai, Xiaozhou Mou

**Affiliations:** 1 General Surgery, Cancer Center, Department of Hepatobiliary & Pancreatic Surgery and Minimally Invasive Surgery, Zhejiang Provincial People’s Hospital, Affiliated People’s Hospital, Hangzhou Medical College, Hangzhou, 310014 China; 2 College of Pharmacy, Hangzhou Medical College, Hangzhou, 310059 China; 3 Clinical Research Institute, Zhejiang Provincial People’s Hospital, Affiliated People’s Hospital, Hangzhou Medical College, Hangzhou, 310014 China; 4 Key Laboratory of Tumor Molecular Diagnosis and Individualized Medicine of Zhejiang Province, Hangzhou, 310014 China; 5 Department of Oncology, Shanghai Medical College, Fudan University, Shanghai, 200032 China; 6 College of Pharmacy, Zhejiang University of Technology, Hangzhou, 310014 China; 7 Zhejiang Healthfuture Biomedicine Co., Ltd., Hangzhou, 310052 China; 8 Department of Dermatology, the First People’s Hospital of Jiashan, Jiaxing, 314100 Zhejiang China

**Keywords:** Engineering stem cell-derived exosomes, Diabetic chronic wounds, Angiogenesis, Optogenetics, Immune microenvironment

## Abstract

**Background:**

Angiogenesis and tissue repair in chronic non-healing diabetic wounds remain critical clinical problems. Engineered MSC-derived exosomes have significant potential for the promotion of wound healing. Here, we discuss the effects and mechanisms of eNOS-rich umbilical cord MSC exosomes (UCMSC-exo/eNOS) modified by genetic engineering and optogenetic techniques on diabetic chronic wound repair.

**Methods:**

Umbilical cord mesenchymal stem cells were engineered to express two recombinant proteins. Large amounts of eNOS were loaded into UCMSC-exo using the EXPLOR system under blue light irradiation. The effects of UCMSC-exo/eNOS on the biological functions of fibroblasts and vascular endothelial cells in vitro were evaluated. Full-thickness skin wounds were constructed on the backs of diabetic mice to assess the role of UCMSC-exo/eNOS in vascular neogenesis and the immune microenvironment, and to explore the related molecular mechanisms.

**Results:**

eNOS was substantially enriched in UCMSCs-exo by endogenous cellular activities under blue light irradiation. UCMSC-exo/eNOS significantly improved the biological functions of cells after high-glucose treatment and reduced the expression of inflammatory factors and apoptosis induced by oxidative stress. In vivo, UCMSC-exo/eNOS significantly improved the rate of wound closure and enhanced vascular neogenesis and matrix remodeling in diabetic mice. UCMSC-exo/eNOS also improved the inflammatory profile at the wound site and modulated the associated immune microenvironment, thus significantly promoting tissue repair.

**Conclusion:**

This study provides a novel therapeutic strategy based on engineered stem cell-derived exosomes for the promotion of angiogenesis and tissue repair in chronic diabetic wounds.

**Graphic Abstract:**

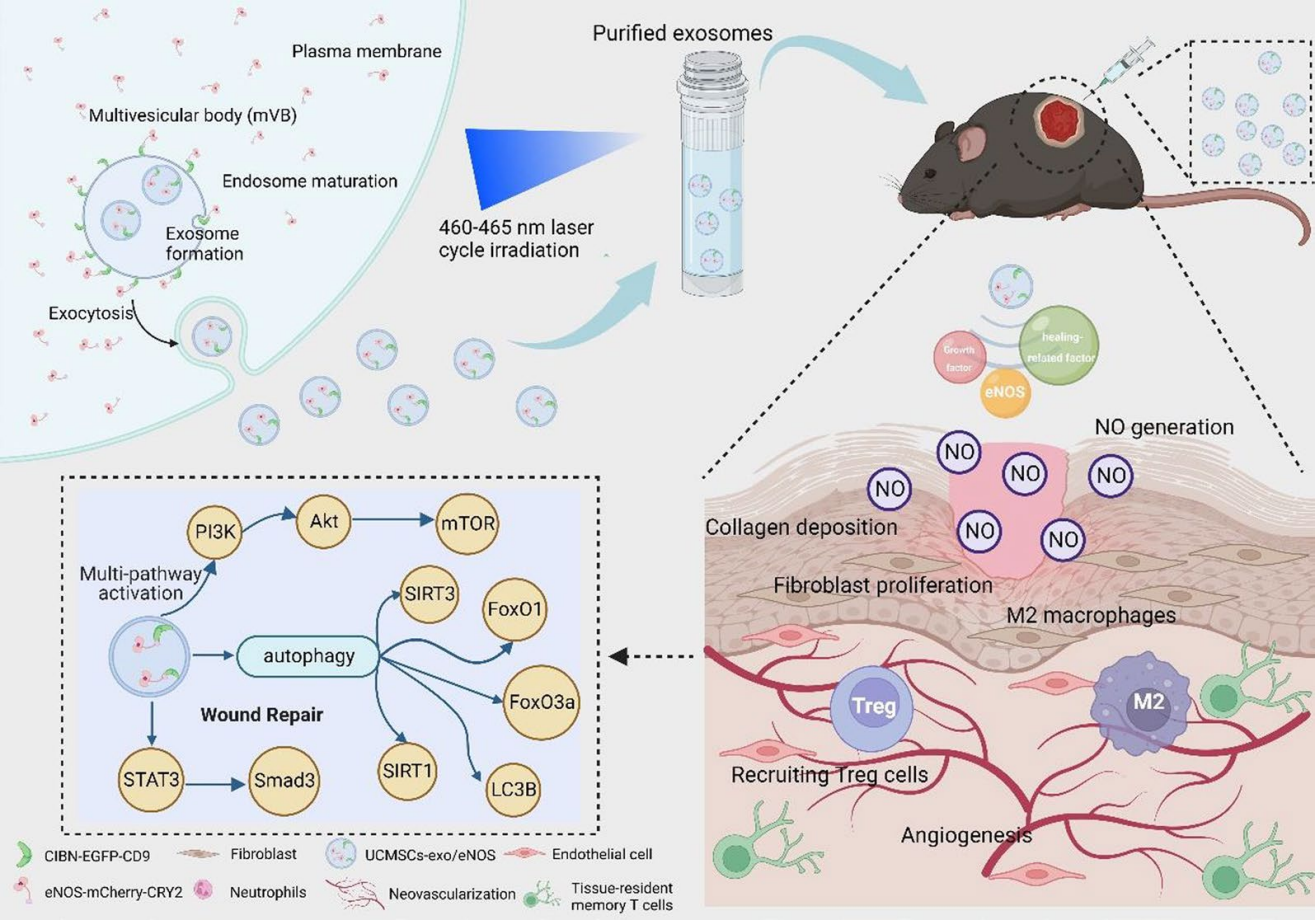

**Supplementary Information:**

The online version contains supplementary material available at 10.1186/s12951-023-01886-3.

## Introduction

Diabetic wounds are one of the common complications of diabetes. Due to the lack of effective treatment strategies, the incidence of delayed wound healing in diabetic patients is increasing year by year [1]. Impaired glucose metabolism in diabetic patients leads to a hyperglycemic environment, which both complicates the various stages of wound healing and is difficult to regulate [2]. Hyperglycemia disrupts a range of biological responses, including impairments in skin cell migration and proliferation at wound sites [3] and the production of healing-related factors [4], as well as promoting the continued production of pro-inflammatory cytokines [5], abnormal angiogenesis [6] and disordered immune responses [7], thus delaying the process of wound healing. Nowadays, conservative treatment of chronic wounds focuses on creating a more favorable local microenvironment for wound healing [8]. Mesenchymal stem cells (MSCs) are known to contribute significantly to angiogenesis and tissue repair, and exosome therapy has been shown to overcome the side effects associated with conventional stem cell transplantation, showing both stability and low immunogenicity [9]. As a cell-free therapy, human umbilical cord mesenchymal cells (UCMSCs)-derived exosomes (UCMSCs-exo) have been shown to promote vascular function and angiogenesis resulting in robust tissue repair [10, 11].

Tissue repair is associated with a complex array of cellular activity and biochemical reactions at the site of injury. A large number of bioregulatory molecules such as nitric oxide (NO) play an indispensable role in wound healing. NO affects collagen remodeling and repairs mechanical strength in wounds [12]. NO synthase (NOS) is one of the enzymes responsible for NO synthesis and the expression of endothelial NO synthase (eNOS) is beneficial to diabetic wound healing [13, 14]. The concentration of eNOS at the wound site affects the wound closure rate, wound breaking strength, and capillary ingrowth [15]. Signaling molecules and cellular responses act either synergistically or in parallel to construct a local microenvironment that is conducive to wound healing [16, 17] and the activation of several signaling cascades contributes to the release of growth factors and angiogenesis in diabetic wounds [18]. The regulation of autophagy during healing from the inflammatory to the remodeling phases has also been found to be effective in improving the healing outcomes of diabetic wounds [19]. Treatment with mesenchymal stem cell exosomes has been shown to enhance both vascular function and angiogenesis in cases where wound-associated endothelial dysfunction has led to impaired vascular function [20] More importantly, exosomes secreted by MSCs can influence macrophage polarization, inhibiting M1 and promoting M2 polarization to reduce the release of pro-inflammatory factors and the inflammatory response. Furthermore, M2 macrophages release pro-angiogenic mediators that promote angiogenesis and collagen deposition [17, 21, 22]. Mesenchymal stem cell exosomes not only improve angiogenesis and molecular activation, but also improve tissue repair through the suppression of inflammation at the wound site and remodeling of the immune microenvironment, including altering the proportions of neutrophils, macrophages, regulatory T cells (Tregs), and memory T cells.

Nowadays, MSCs-derived exosomes are used not only as carriers with inherited homing ability to target the site of injury [23], but are also applied as biologicals to enhance angiogenesis and tissue remodeling [24]. It is also possible to enhance the biological functions of exosomes by drug stimulation [25, 26], genetic engineering [27], or surface modification [28]. In recent years, the EXPLOR system has been developed based on the novel concept of utilizing genetic engineering and optogenetic techniques to mediate interactions with protein modules and enrich exosome cargoes with specific target proteins using endogenous biological secretions [29]. This allows the stable transportation of therapeutic macromolecular proteins in exosomes without the need for protein isolation and purification, as well as avoiding potentially uncontrollable biological problems, thus providing a new direction in exosome-based protein therapy.

In this study, we used UCMSCs-exo as a therapeutic vector to deliver eNOS to the injury target. eNOS were spontaneously loaded into UCMSC-derived exosomes by EXPLOR, a blue light-mediated reversible protein–protein interaction technique. For stable exosome production, we constructed UCMSCs expressing two recombinant proteins, CIBN-EGFP-CD9 and eNOS-mCherry-CRY2. The application of the purified eNOS-enriched UCMSCs-exo for the treatment of chronic diabetic wounds led to significant improvements in wound-site angiogenesis and collagen remodeling while inhibiting neutrophil infiltration by the alleviation of chronic inflammation, remodeling of the macrophage anti-inflammatory phenotype, and the recruitment of Tregs to the enriched wound site to modulate the local immune microenvironment, thereby accelerating tissue repair. In conclusion, this study provides a novel strategy to explore the enhanced effects of engineered mesenchymal stem cell-derived exosomes for the promotion of angiogenesis and tissue repair in chronic diabetic wounds.

## Methods

### Animals

Male C57BLKS-Leprdb (db/db) mice (8 weeks old) were purchased from GemPharmatech Co., Ltd. All mice were housed in a specific pathogen-free area and were maintained in a 12-h light: 12-h dark cycle with controlled temperature (24 °C ± 1 °C) and relative humidity (50–60%). All the mice had free access to food and water. All surgical procedures and methods used in the study were approved by the Ethics Committee of Zhejiang Provincial People's Hospital (No. A20220015).

To establish the model of chronic diabetic skin wounds, the mice were anesthetized with 4% (vol/vol) isoflurane (RWD Life Science, USA) before the operation. After the animals were shaved, a circular full-thickness wound of 1.5 cm in diameter was created on the back of each mouse. The wound site was observed daily and was photographed using a digital camera on days 0, 7, 14, and 21. ImageJ software was used to measure and calculate the wound dimensions.

After surgery, UCMSCs-exo/eNOS or UCMSCs-exo (20 μg dissolved in 100 μL phosphate-buffered saline) or an equal volume of phosphate-buffered saline (PBS) were injected subcutaneously into the wound every other day. On the indicated day, skin tissue surrounding the wound was collected for analysis.

### Culture of fibroblasts and endothelial cells

Human vascular endothelial cells (HUVECs) and L929 mouse skin fibroblasts were purchased from the Chinese Academy of Sciences (Shanghai, China) and cultured in Dulbecco’s Modified Eagle Medium (DMEM) supplemented with 10% FBS (Biological Industries, USA) and 1% penicillin–streptomycin (Thermo Fisher Scientific, USA). Cells were incubated at 37 °C with 5% CO_2_.

### Tubule formation assay

To study capillary-like construction activity in HUVEC cells, the formation of vascular-like structures was assessed using human fibrin matrices (Corning, USA). In brief, cold Matrigel (250 µL per well) was pipetted into 24-well plates using a pre-cooled pipette tip. This was followed by the addition of 400 μL of a suspension of 3,3-Dioctadecyloxacarbocyanine perchlorate (DiO)-stained HUVECs (7.5 × 10^5^ cells/well, untreated or treated with UCMSCs-exo/eNOS or UCMSCs-exo) on the Matrigel, and incubated at 37 °C for 12 h. Tube-forming capacity was assessed by observing the tubular structures using a fluorescence microscope (Olympus, Japan).

### Reactive oxygen species (ROS) detection

Intracellular ROS production was determined by 2′,7′-Dichlorofluorescin diacetate (DCFH-DA) staining (Beyotime, China). Reactive oxygen species (ROS) in cells can oxidize non-fluorescent DCFH to produce fluorescent DCF, and the fluorescence intensity of DCF can thus reflect the ROS level. Fibroblasts or endothelial cells were seeded in 6-well plates at a density of 2 × 10^5^ cells/well. After reaching 80% confluence, the cells were incubated with UCMSCs-exo/eNOS or UCMSCs-exo for 24 h and then subjected to high-glucose (HG, 50 mM) or H_2_O_2_ (200 μM) stimulation. After the indicated times, cells were incubated with 10 μm DCFH-DA for 20 min at 37 °C in serum-free medium and then washed three times with PBS. The probe-loaded cells were observed by confocal laser microscopy and the fluorescence intensity was analyzed by ImageJ software.

### Mitochondrial membrane potential measurement (JC-1 staining)

The mitochondrial membrane potential was detected by JC-1 staining (Beyotime, China). JC-1 is an ideal fluorescence probe that is widely used for the determination of the mitochondrial membrane potential. When the mitochondrial membrane potential is high, JC -1 accumulates in the mitochondrial matrix and emits red fluorescence. At low mitochondrial membrane potentials, JC-1 produces green fluorescence in the presence of the monomer. Fibroblasts or endothelial cells were cultured in 6-well plates at densities of 2 × 10^5^ cells per well and incubated with UCMSCs-exo/eNOS or UCMSCs-exo for 24 h. When the cells reached 80% confluence, they were treated with hydrogen peroxide (200 μM) for 12 h. The JC-1 working solution was prepared according to the manufacturer's instructions and was added to the wells and incubated at 37° C for 20 min. After three washes with buffer, the changes in fluorescence were monitored by fluorescence microscopy and the ratio of red to green fluorescence intensity was analyzed using ImageJ.

### TUNEL

Apoptosis of endothelial cells treated with H_2_O_2_ (200 μM) for 12 h was assessed using a fluorescent TUNEL detection kit (Beyotime, China), according to the manufacturer's instructions. The cells were fixed with 4% paraformaldehyde at room temperature for 20 min, followed by permeabilization with 0.3% Triton X-100 for 5 min, and the addition of the TUNEL detection solution. The cells were incubated at 37 °C for 60 min in the dark. The nuclei were counterstained with DAPI. TUNEL-positive cells were evaluated and counted under confocal microscopy.

### Histological analysis

The skin of the wound tissue was collected on postoperative days 7, 14, and 21, fixed with 4% paraformaldehyde, and embedded in paraffin after dehydration. The paraffin-embedded tissues were sliced into 5-μm-thick sections. Re-epithelialization and the degree of collagen maturation were observed by hematoxylin and eosin (H&E) or Masson’s trichrome staining. Images were examined under an optical microscope (Olympus, Japan).

For immunohistochemical staining, the paraffin sections were rehydrated and incubated with the primary antibody, followed by incubation with the secondary antibody and the streptavidin biotin–peroxidase complex. Finally, the samples were visualized by the chromogenic substrate diaminobenzidine (DAB). The stained sections were observed with an optical microscope.

For the assessment of immunofluorescence, the paraffin sections were rehydrated, blocked with 1.5% goat serum, and incubated with the primary antibody overnight at 4 °C. The sections were then treated with Alexa Fluor 488 and Cy3-conjugated secondary antibodies, while the nuclei were stained with DAPI. The sections were examined and imaged using a confocal microscope and the fluorescence area and intensity were evaluated by ImageJ software.

### Quantitative real-time PCR (qRT-PCR)

Total RNA was extracted with TRIzol reagent and complementary DNA (cDNA) was obtained by the reverse transcription of 1 μg of total RNA from each extracted sample using the PrimeScript RT reagent kit (Takara Biotechnology, Japan). Next, the SYBR Green detection reagent (Takara Biotechnology) was used for qRT-PCR analysis in an Applied Biosystems 7500 Real-Time PCR System (Applied Biosystems, USA). Beta-actin or GAPDH was used for normalization of the results. The sequences of the primers used in this study are provided in Additional file 4: Table S1.

### Western blotting

Briefly, tissues were homogenized and lysed on ice for 30 min in pre-chilled RIPA buffer containing a phosphatase inhibitor cocktail and PMSF. The lysates of exosomes or tissues were diluted in a 1:5 ratio with protein loading buffer (5 ×) (ABclonal, China) and heated at 95 °C for 5 min. Protein extracts were separated on 4‒20% sodium dodecyl sulfate–polyacrylamide gel electrophoresis (SDS-PAGE) gels at 120 V and blotted at 220 mA onto polyvinylidene di-fluoride (PVDF) membranes (Merck Millipore, Germany) for 90 min. The membranes were blocked with Blocking Buffer (Yoche, China) for 10 min at room temperature followed by overnight incubation at 4 °C with the primary antibodies. The following day, the membranes were incubated with horseradish peroxidase (HRP)-linked secondary antibodies (HuaBio, China) for 2 h at room temperature. Finally, protein bands were visualized using an ECL substrate kit (Bio-Rad, USA) and the expression levels of the proteins were quantified by ImageJ. All primary antibodies were from Cell Signaling Technology (USA). All protein expression was normalized to β-actin or GAPDH.

### Culture and identification of human UCMSCs

Human umbilical cord-derived mesenchymal stem cells were provided by Weiwei Biomedical Technology (China) and their identification was completed with the full support of Weiwei Biomedical Technology. The UCMSC cell line was cultured in MesenCult™ MSC Basal Medium containing MesenCult™ MSC Stimulatory Supplemen (STEMCELL Technologies, Canadian). UCMSC surface marker proteins were evaluated by flow cytometry (Agilent, USA); these included three positive markers (CD90, CD105, and CD73), six negative cocktails (CD45, CD34, CD14, CD11b, CD19, and HLA-DR), and the respective isotype controls. All antibodies were obtained from BD Biosciences (San Jose, CA, USA). Osteogenesis, adipogenesis, and chondrogenesis of UCMSCs were evaluated using the MesenCult™ osteogenic, adipogenic, and chondrogenic differentiation kit (STEMCELL Technologies). Differentiation properties were identified according to the manufacturer's protocol.

### UCMSCs-exo/eNOS isolation and identification

The recombinant type 5 adenovirus expressing two fusion proteins, CIBN-EGFP-CD9 and eNOS-mCherry-CRY2 was contracted for production to Shanghai Genechem Co., Ltd. To upregulate CIBN-EGFP-CD9 and eNOS-mCherry-CRY2 for protein–protein interactions using optogenetics, the adenovirus vectors carrying the fusion protein CIBN-EGFP-CD9 or eNOS-mCherry-CRY2 were transfected into UCMSCs according to the manufacturer’s protocol.

Ultracentrifugation was used for the isolation and extraction of exosomes. UCMSCs overexpressing CIBN-EGFP-CD9 and eNOS-mCherry-CRY2 were seeded into T175 flasks. After one day, the medium was carefully removed, and exosome-depleted medium was added.

Then, the cells were exposed to continuous blue light illumination from a 460: 465 nm light board in a CO_2_ incubator. After 72 h, the cell culture supernatant was harvested and centrifuged at 300*g* for 10 min and 2000*g* for 30 min to remove dead cells and cellular debris. After further centrifugation at 10 000*g* for 30 min, the supernatant was filtered through a 0.22-µm filter (Merck-Millipore). The supernatant was then centrifuged twice at 100 000*g* for approximately 1.5 h each. The pellets were resuspended in PBS and stored at −80 °C for further experiments. The separation and purification of UCMSCs-exo was performed using the same procedures.

To verify the ultrastructure and shape of the exosomes, the exosomes were evaluated using transmission electron microscopy (TEM). Dynamic light scattering (DLS, Malvern Instruments, UK) was used for determining the size distribution and particle concentration of the exosomes. The presence of specific exosomal surface markers (TSG101, CD9, CD63, CD81) was evaluated using Western blotting.

### Exosome labeling and uptake

Exosomes were labeled with the Dil fluorescent labeling kit (Yeasen, China). Dil (10 μm) was added to an exosome suspension and incubated at room temperature for 20 min in the dark. Exosomes were collected by centrifugation at 100 000*g* for 90 min and then washed twice with PBS to remove any unbound dye. The Dil-labeled exosomes were then incubated with the HUVECs for 24 h. The cells were then fixed and the nuclei were stained with Hoechst. Images were obtained by confocal microscopy.

### Lipid peroxidation determination

Lipid peroxidation was assessed by measuring the red byproduct of the reaction of malondialdehyde (MDA) with thiobarbituric acid (T BA). Cells were treated with UCMSCs-exo/eNOS or UCMSCs-exo for 24 h and exposed to high glucose (50 mM) for 24 h. Absorbances were measured and standard curves were established in accordance with the manufacturer's instructions (Beyotime, China).

### SOD determination

The activity of superoxide dismutase (SOD) was determined by the WST-8 method. WST-8 can react with xanthine oxidase-catalyzed superoxide anion radicals to produce water-soluble formazan dye and as SOD can inhibit formazan dye production through dissimilation, the activity of SOD can be measured by colorimetric analysis. SOD activities were measured in the cells after different treatments according to the manufacturer's instructions (Beyotime, China).

### Total glutathione assay

This was measured by the reduction of oxidized glutathione (GSSG) to reduced glutathione (GSH) by the mitochondrial enzyme glutathione reductase. GSH reacts with the chromogenic substrate DTNB to produce yellow TNB and GSSG. The total glutathione content was calculated by adjusting the reaction system and measuring the amount of yellow TNB formed. Each group of cells was measured after 24 h of high-glucose stimulation, according to the manufacturer's instructions (Beyotime, China).

### NO measurement

NO is easily oxidized to NO_2_- and NO_3_- in vivo and NO_3_- is reduced to NO_2_- by nitrate reductase. Under acidic conditions, NO_2_- forms diazo compounds with diazosalt sulfonamide, which can be further coupled to naphthyl vinyl diamine. The product had a characteristic absorption peak at 550 nm, allowing calculation of the NO content by measuring the absorption value. Skin tissue was collected and homogenized. According to the manufacturer's instructions, the Micro NO Content Assay Kit (Solarbio, China) was used for NO measurements.

### Transwell migration assay

Cell migration was measured by the Transwell method using 24-well Transwell culture plates (Corning, USA) with 8 μm pore-sized filters was used. Approximately 1 × 10^4^ cells were inoculated into the upper chamber and incubated with serum-free medium. Then, different treatments containing HG (50 mM), UCMSCS-exo (20 μg/ml), and UCMSCs-exo/eNOS (20 μg/ml) were added to the lower chamber. Cells were cultured for 24 or 48 h in an incubator at 37 °C, after which the medium was removed, and cells were washed three times with PBS. Subsequently, cells were fixed with 4% paraformaldehyde for 15 min and then stained with 0.1% crystal violet for several minutes. A cotton swab was used to remove cells from the top surface of the filter. Migratory activity was assessed by observing the stained cells under an optical microscope and their enumeration using ImageJ software.

### Cell migration assays

Cells (2 × 10^5^ cells per well with three replicates per group) were seeded in 6-well plates and incubated at 37 °C. After the cells reached confluency, the monolayer was manually scratched with the tip of pipette, and the detached cells were removed by washing with serum-free medium. The cells were then cultured in medium supplemented with or without HG (50 mM), UCMSCS-exo (20 μg/ml), and UCMSCs-exo/eNOS (20 μg/ml). All cells were treated with Mitomycin-C for 1 h before scratching to exclude the influence of cell proliferation on wound closure. The matched wound areas were photographed at 0, 24, 48, and 72 h after wounding. The migration area (%) was calculated as (A0-An)/A0 × 100%, where A0 represents the initial wound area and An represents the remaining area of the wound at the measured time point.

### Proliferation assay

Briefly, cells (5 × 10^3^ cells per well, six replicates per group) were seeded into 96-well culture plates and treated with UCMSCs-exo (20 μg/ml) or UCMSCs-exo/eNOS (20 μg/ml). The cell-free group served as the blank group. At 12, 24, 36, and 48 h, the Cell Counting Kit-8 reagent (CCK-8, Yeasen, China; 20 μL per well) was added to the medium (100 μL per well). After incubation at 37 °C for 2 h, the absorbance of each well was measured at 450 nm by a microplate reader. The OD values were used to assess cell proliferation.

### Calcein/PI cell viability/cytotoxicity assay

The survival and death of cells were assessed using Calcein-AM (Calcein-AM) and PI (propidium iodide) double-fluorescence staining (Beyotime, China). In brief, approximately 2 × 10^5^ HUVECs were seeded in 6-well plates and incubated in complete medium with or without UCMSCs-exo/eNOS (20 μg/ml) and UCMSCs-exo (20 μg/ml) for 24 h before stimulation with H_2_O_2_ for 12 h. The calcein AM/PI detection solution was added and incubated for 30 min at 37 °C in the dark according to the manufacturer's instructions. The nuclei were stained with Hoechst. The cells were visualized by confocal microscopy.

### Flow cytometry

The attached adipose tissue was removed before harvesting the skin wound tissue and the skin samples were sliced and digested with the Opti-MEM (Invitrogen) solutions containing collagenase type I (0.5 mg/ml, BioFroxx, China), collagenase type II (0.5 mg/ml, BioFroxx, China), collagenase type IV (1 mg/ml, BioFroxx, China), hyaluronidase (1 mg/ml, BioFroxx, China), and deoxyribonuclease I (0.02 mg/ml, Biosharp, China) for 30 min on a shaker at 37 °C. The tissue was mechanically ground using a Tissue Grinder with Pestle (YiXi Bio, China), washed with a complete medium containing 10% FBS, and the red blood cells were lysed.

Cell surfaces were stained in the dark on ice for flow cytometry analysis. The cells were stained with antibodies against the surface antigens CD4 (MultiSciences, China), CD25 (MultiSciences, China), CD8 (Proteintech, China), CD69 (Proteintech), and CD103 (Proteintech). Cells were fixed and permeabilized and then incubated with anti-CD16/32 (MultiSciences, China) to block nonspecific binding. Staining of intracellular markers, such as the Treg marker FoxP3 (MultiSciences, China), were performed on ice. The cells were then analyzed by flow cytometry.

### Statistical analysis

All data are presented as means ± standard deviation (SD). Independent-sample t-tests were used to compare the means between two different groups. One-way analysis of variance (ANOVA) was utilized for the evaluation of the significant difference. Multiple-group comparisons were performed using one-way ANOVA. GraphPad Prism 8 software was used for all statistical analyses. *P*-values < 0.05 were considered statistically significant.

## Results

### Construction of genetically engineered UCMSCs-eNOS and isolation and characterization of optogenetic exosomes UCMSCs-exo/eNOS

**Fig. 1:**
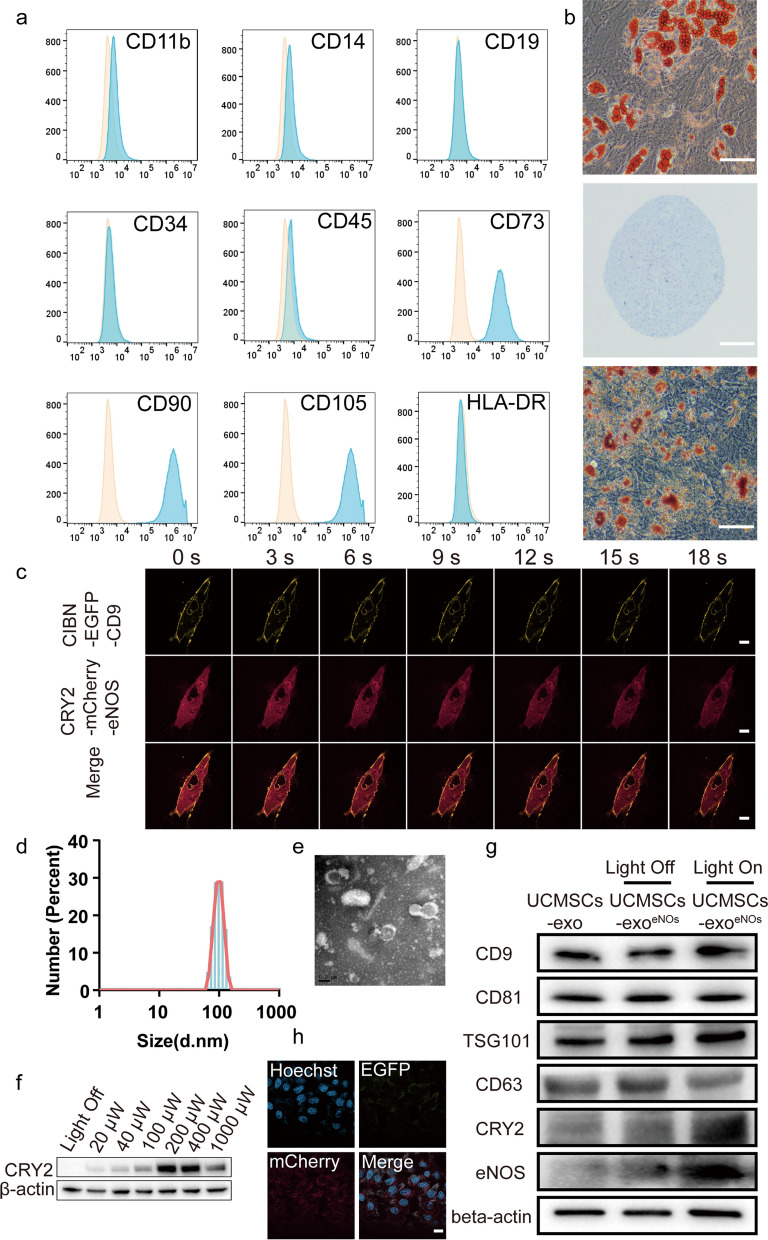
Construction and characterization of genetically and optogenetically engineered UCMSCs and UCMSCs-exo/eNOS. **a** Flow cytometry analysis of cell surface markers on UCMSCs. The isotype control is illustrated as an orange curve and the test samples are illustrated as solid blue curves. **b** Tri-lineage differentiation assay of UCMSCs. UCMSCs were able to differentiate into adipocytes, chondrocytes, or osteoblasts when cultured in lipogenic, chondrogenic, or osteogenic media as indicated by Alizarin Red S staining (scale bar: 400 μm), Oil Red O staining (scale bar: 400 μm), and Alcian Blue staining (scale bar: 400 μm). **c** Imaging of UCMSCs expressing two recombinant proteins, CIBN-EGFP-CD9 and eNOS-mCherry-CRY2, before and after laser stimulation at 488 nm. Scale bar: 20 μm. **d** Size distribution of UCMSCs-exo/eNOS obtained using dynamic light scattering. The average width of the UCMSCs-exo/eNOS was 78.82 nm. **e** Morphology of USC-Exos observed by transmission electron microscopy. Scale bar: 200 nm. **f** UCMSCs expressing CIBN-EGFP-CD9 and eNOS-mCherry-CRY2 were cultured under blue light irradiation at different powers for 48 h. The isolated exosomes were analyzed by immunoblotting with an antibody against CRY2. **g** Lysis and immunoblot analysis of UCMSCs and UCMSCs^eNOS^-secreted exosomes. **h** Confocal microscopy analysis showing DiI-labeled UCMSCs-exo/eNOS incorporated into human vascular endothelial cells (HUVECs). Scale bar: 20 μm. All experiments were repeated three times independently

Human umbilical cord mesenchymal stem cells (UCMSCs) are present in umbilical cord tissue and have self-renewal and pluripotent properties. In accordance with the International Society for Cellular Therapy position statement, we identified the collected UCMSCs by MSC marker staining and flow cytometry. The UCMSCs were found to be strongly positive for the surface markers CD73, CD90, and CD105 but negative for the CD11b, CD14, CD19, CD34, CD45, and HLA-DR surface markers (Fig. 1a). To determine the pluripotency of the UCMSCs, a tri-lineage differentiation assay was performed (Fig. 1b) by inducing UCMSCs in vitro with osteogenic, chondrogenic, or lipogenic cultures. Alizarin red staining was used to assess amorphous calcium salt deposition and to observe osteogenesis. Chondrogenesis was investigated by examining the presence of chondrocyte-associated polysaccharides and proteoglycans using Alcian blue staining while Oil Red O staining was used to detect the formation of intracellular lipid droplets in adipocytes. The results confirmed that the UCMSCs has the characteristics and multidirectional differentiation potential of MSCs.

To produce stem cell-derived exosomes with efficient loading of eNOS proteins, we constructed UCMSCs that overexpressed two fusion proteins, CIBN-EGFP-CD9 and eNOS-mCherry-CRY2 (UCMSCs^eNOS^). Laser irradiation at 488 nm with laser confocal microscopy induced the rapid migration of eNOS-mCherry-CRY2 from the cytoplasm to the cell membrane and intracellular compartments to conjugate with CIBN-EGFP-CD9, demonstrating co-localization of EGFP with mCherry (Fig. 1c) and indicating that the EXPLORs system was feasible for construction in stem cells. Due to the difficulties involved in the preparation of large LED light plates at specific wavelengths, we chose lasers with similar wavelengths to construct the optical equipment, which also had the ability to induce interaction between the two photosensitive proteins, as shown by previous studies [30–32]. We placed LED light panels with emission wavelengths in the range of 460–465 nm (light frequency of 50 Hz and maximum working light power of 2 mW) in a constant temperature incubator. After irradiation at a distance of 10 cm from the cells for 48 h (the light cycle consisted of one minute of blue light irradiation and one minute of darkness over a period of 48 h), the supernatant was collected and the exosomes were isolated. Dynamic light scattering showed that there was a size concentration distribution around 78.82 nm (Fig. 1d). Transmission electron microscopy revealed an intact bilayer membrane structure (Fig. 1e). To determine the optimal production conditions, we performed immunoblotting of exosomes from different treatments and found that the amount of functional protein, eNOS-mCherry-CRY2, from optogenetically engineered UCMSCs grown under blue light irradiation was significantly higher than the amount of eNOS-mCherry-CRY2 protein obtained from cells grown in the dark, and that the greatest efficiency of eNOS-mCherry-CRY2 transport occurred in the power range of 200 to 400 μW (Fig. 1f). This corresponds to the pre-exploration of the EXPLORs system constructed by Hojun Choi et al. [29]. The exosomal biomarkers CD9, CD81, TSG101, and CD63 were observed in the samples, which demonstrated that this optogenetically engineered exosome maintained its exosomal characteristics. We also found that eNOS-mCherry-CRY2 was heavily loaded into exosomes derived from UCMSCs^eNOS^ cells (Fig. 1g). We co-incubated the UCMSC-derived exosomes loaded with eNOS-mCherry-CRY2 (UCMSCs-exo/eNOS) with HUVECs to verify the uptake of UCMSCs-exo/eNOS. UCMSCs-exo/eNOS carrying both fluorescent proteins were found in the HUVEC cytoplasm (Fig. 1h).

### UCMSCs-exo/eNOS enhances the biological functions of fibroblasts and vascular endothelial cells

**Fig. 2:**
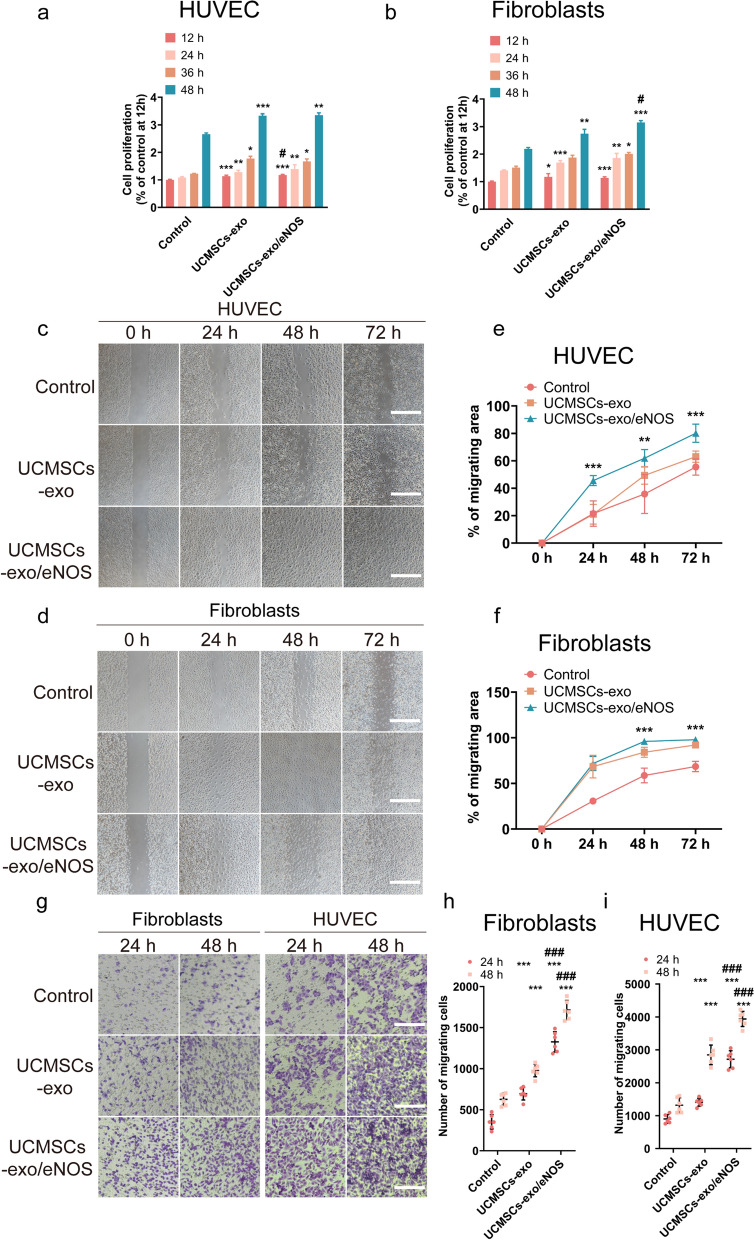
UCMSCs-exo/eNOS promote proliferation and migration of fibroblasts and vascular endothelial cells. **a** Proliferation of HUVECs incubated in complete medium supplemented with UCMSCs-exo and UCMSCs-exo/eNOS for 12, 24, 36, and 48 h. Each group was set up with three replicates. **b** Proliferation of fibroblasts incubated in complete medium supplemented with UCMSCs-exo and UCMSCs-exo/eNOS for 12, 24, 36 and 48 h. Each group was set up with three replicates. **c** Scratch assay to assess the migration of HUVEC treated with UCMSCs-exo and UCMSCs-exo/eNOS. Representative pictures of wound closure at 0, 24, 48, and 72 h with UCMSCs-exo vs. UCMSCs-exo/eNOS vs. Control group. Scale bar: 200 μm. **d** Scratch assay to assess migration of fibroblasts treated with UCMSCs-exo and UCMSCs-exo/eNOS. Representative images of wound closure at 0, 24, 48, and 72 h with UCMSCs-exo vs. UCMSCs-exo/eNOS vs. Control group. Scale bars: 200 μm. **e** Graph showing quantification of HUVEC wound closure. Each group was set up with six replicates. **f** Graph showing quantification of fibroblast wound closure. Each group was set up with 6 replicates for validation. **g** Representative micrographs showing the effect of UCMSCs-exo vs. UCMSCs-exo/eNOS on Transwell migration of fibroblasts and HUVECs. Scale bar: 200 μm. **h** Quantitative analysis of Transwell assay of fibroblasts. Each group was set up with six replicates. **i** Quantitative analysis of Transwell assay of HUVECs. Each group was set up with six replicates. Data represent means ± SD. *p < 0.05, **p < 0.01, ***p < 0.001 vs. Control group; ^#^*p* < 0.05, ^##^*p* < 0.01, ^###^*p* < 0.001 vs. UCMSCs-exo group (two-tailed Student’s t-test)

Fibroblasts and endothelial cells play important roles in the process of chronic wound healing. After treatment of fibroblasts and HUVECs with UCMSCs-exo or UCMSCs-exo/eNOS, respectively, we found that both UCMSCs-exo and UCMSCs-exo/eNOS enhanced endothelial cell and fibroblast viability, while UCMSCs-exo/eNOS showed better proliferation-promoting ability at specific time points (Fig. 2a, b). The scratch wound assay (Fig. 2c, d) and Transwell assays (Fig. 2g) showed that HUVECs and fibroblasts treated with UCMSCs-exo or UCMSCs-exo/eNOS exhibited better migratory abilities compared with the Control group. In addition, treatment with the same dose of UCMSCs-exo/eNOS resulted in greater migration, demonstrating a stronger ability to promote migration (Fig. 2e, f, h, i).

### UCMSCs-exo/eNOS enhanced fibroblast and vascular endothelial cell resistance to inhibition of biological functions and peroxidation induced by high glucose.

**Fig. 3:**
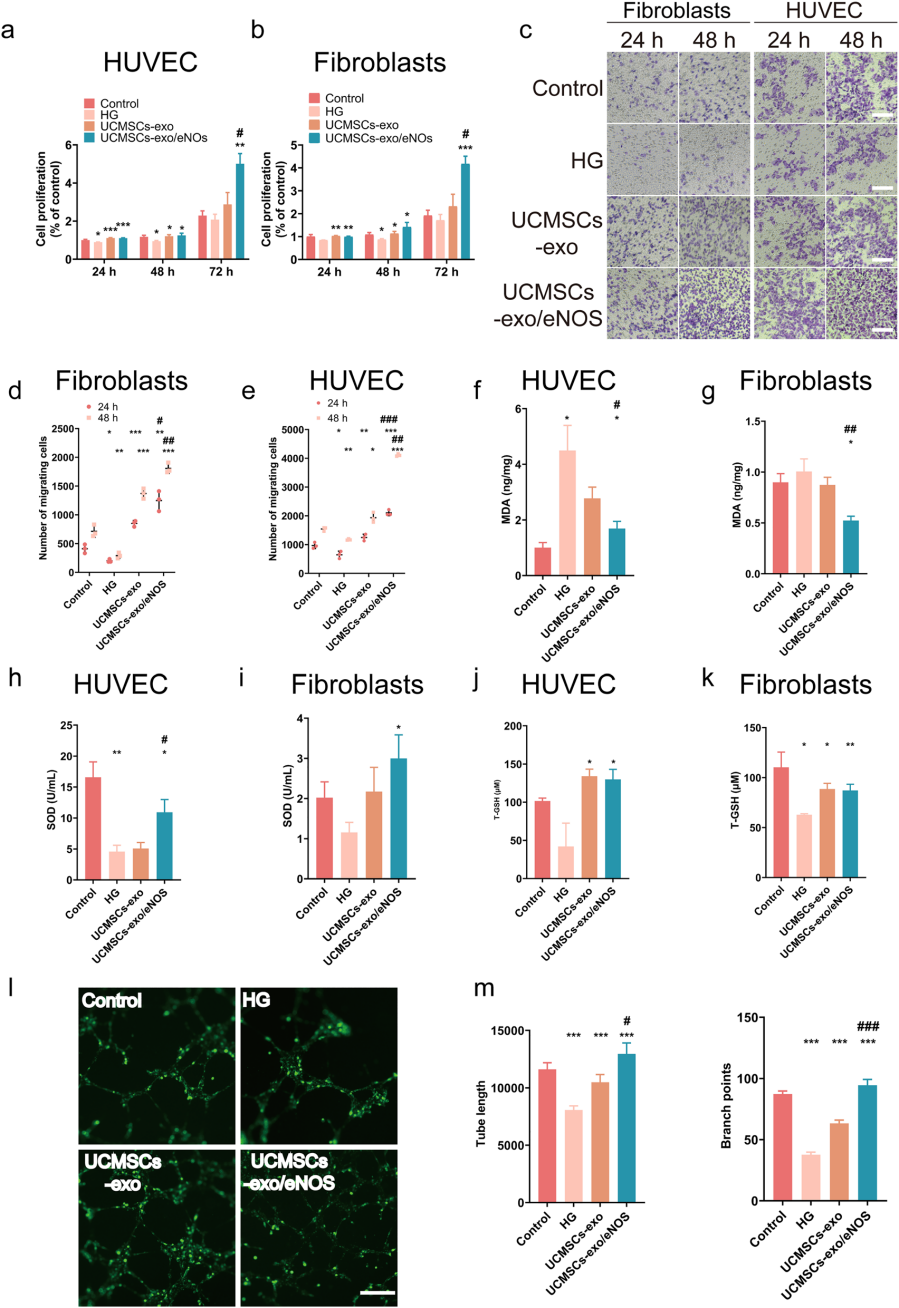
UCMSCs-exo/eNOS counteract oxidative damage and restore biological functions in fibroblasts and vascular endothelial cells treated with high glucose. HG: complete medium containing 50 mM glucose. UCMSCs-exo: supplemented with 20 μg of UCMSCs-exo in complete medium containing 50 mM glucose. UCMSCs-exo/eNOS: supplemented with 20 μg of UCMSCs-exo/eNOS in complete medium containing 50 mM glucose. **a** Proliferation of HUVECs incubated with HG, UCMSCs-exo, and UCMSCs-exo/eNOS for 12, 24, 36 and 48 h (n = 3, two-tailed Student’s t-test). **b** Proliferation of fibroblasts incubated with HG, UCMSCs-exo, and UCMSCs-exo/eNOS for 12, 24, 36, and 48 h (n = 3, two-tailed Student’s t-test). **c** Representative micrographs showing the effect of UCMSCs-exo versus UCMSCs-exo/eNOS on Transwell migration of fibroblasts and HUVECs in high-glucose culture conditions. Scale bar: 200 μm. **d** Quantitative analysis of Transwell assay of fibroblasts (n = 3, two-tailed Student’s t-test). **e** Quantitative analysis of Transwell assay of HUVECs (n = 3, two-tailed Student’s t-test). **f**, **g** Histograms showing malondialdehyde (MDA) contents of HUVECs and fibroblasts in the Control, HG, UCMSCs-exo, and UCMSCs-exo/eNOS groups (n = 3, two-tailed Student’s t-test). **h**, **i** Histograms showing superoxide dismutase (SOD) activities in HUVECs and fibroblasts in the Control group, HG group, UCMSCs-exo group, and UCMSCs-exo/eNOS group (n = 3, two-tailed Student’s t-test). **j**, **k** Histograms showing the glutathione (GSH) contents of HUVECs and fibroblasts in the Control group, HG group, UCMSCs-exo group, and UCMSCs-exo/eNOS group (n = 3, two-tailed Student’s t-test). **l** Representative images of tube formation assays of HUVECs treated with HG, UCMSCs-exo, or UCMSCs-exo/eNOS on Matrigel. Scale bar: 200 μm. **m** Quantitative analysis of tube lengths and branching points in tube formation assays. (Each group was set up with three replicates, and no fewer than six outcomes per group were included in the statistics. Mann–Whitney U test). Data represent means ± SD. *p < 0.05, **p < 0.01, ***p < 0.001 vs. HG group; #*p* < 0.05, ##*p* < 0.01, ###*p* < 0.001 vs. UCMSCs-exo group

To verify the resistance of UCMSCs-exo/eNOS to the damage caused by high-glucose conditions, we treated fibroblasts and HUVECs that had been cultured in high-glucose environments with UCMSCs-exo and UCMSCs-exo/eNOS. The results showed that both UCMSCs-exo and UCMSCs-exo/eNOS restored the HG-mediated damage and promoted the viability of both endothelial cells and fibroblasts, with UCMSCs-exo/eNOS showing stronger pro-proliferative effects than any group at 72 h (Fig. 3a, b). Both HUVECs and fibroblasts treated with UCMSCs-exo/eNOS showed increased migration at all time points tested (Additional file 1: Figure S1a–d and Fig. 3c–e). High concentrations of glucose result in the production and accumulation of excess reactive oxygen species (ROS), a key mediator of cell damage, and we found that UCMSCs-exo/eNOS significantly reduced the excess ROS production induced by HG (Additional file 1: Figure S1e). Furthermore, UCMSCs-exo/eNOS were better able to counteract MDA production induced by high glucose-induced lipid peroxidation and alleviate the levels of oxidative stress in cells (Fig. 3f, g). This was also demonstrated by the recovery of SOD and T-GSH activities in HUVECs and fibroblasts (Fig. 3h–k). Tubule formation assays were performed to determine the pro-angiogenic capacity of UCMSCs-exo/eNOS. After co-incubation with UCMSCs-exo/eNOS for 12 h, greater numbers of capillary-like structures were observed on the Matrigel. In terms of promoting angiogenesis, UCMSCs-exo/eNOS were more effective than UCMSCs-exo, resulting in greater enhancement of tubule length and degree of branching in the treated HUVECs (Fig. 3l, m).

### UCMSCs-exo/eNOS reverse oxidative stress-mediated damage to vascular endothelial cells

**Fig. 4:**
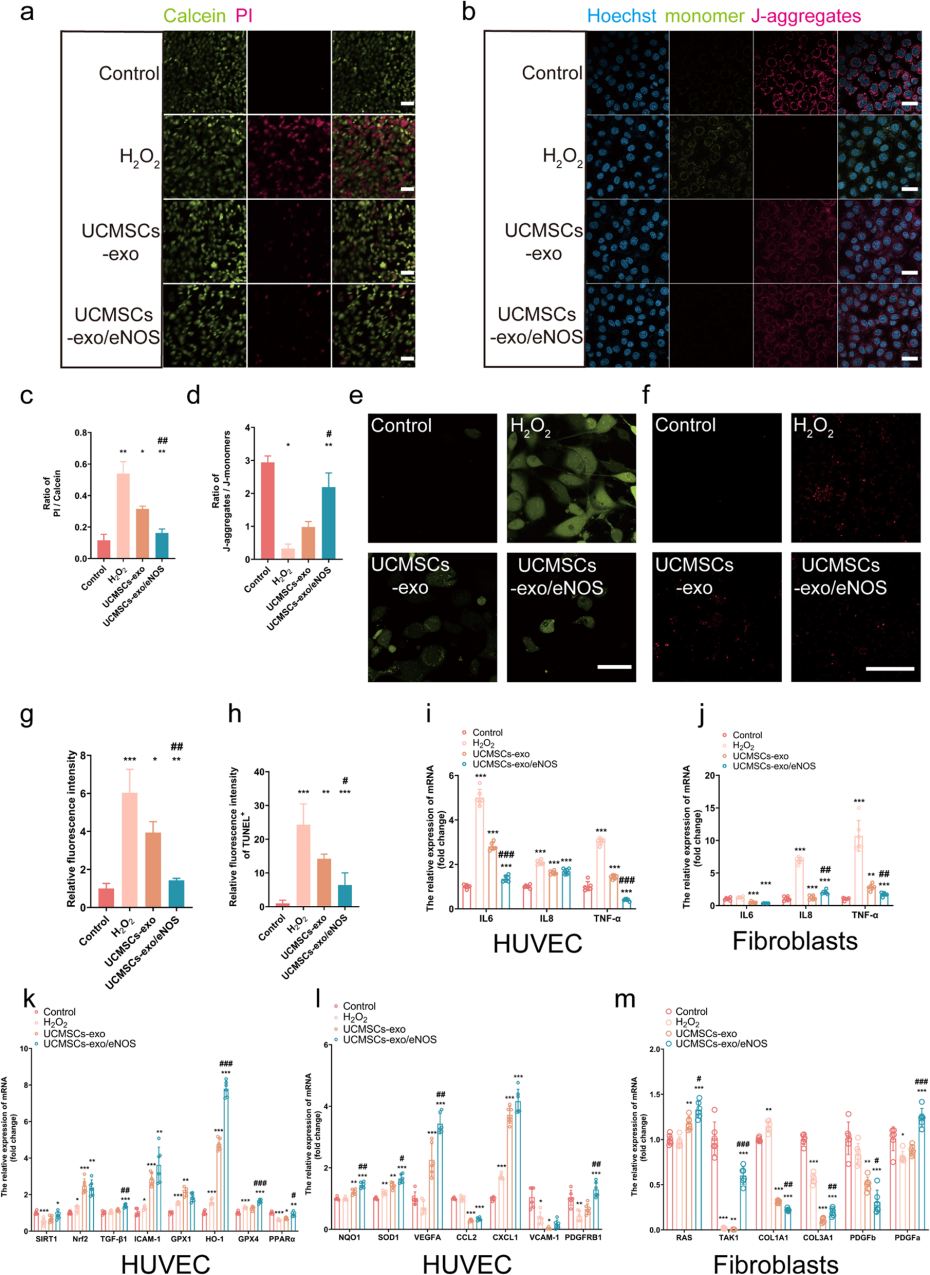
UCMSCs-exo/eNOS enhance the resistance of vascular endothelial cells to oxidative stress and cellular damage brought about by hydrogen peroxide. H_2_O_2_: stimulation with 200 μM hydrogen peroxide. UCMSCs-exo: stimulation with 200 μM hydrogen peroxide after 24 h incubation in complete medium supplemented with 20 μg of UCMSCs-exo. UCMSCs-exo/ eNOS: received 200 μM hydrogen peroxide after 24 h of incubation in complete medium supplemented with 20 μg of UCMSCs-exo/eNOS. **a** Live cells are labeled by Calcein (green fluorescence) and dead cells are labeled by PI (red fluorescence). Examples of the activity profile of HUVECs after stimulation with H_2_O_2_, UCMSCs-exo or UCMSCs-exo/eNOS. **b** Representative plot of mitochondrial membrane potential shown by JC-1 staining of HUVECs cultured with H_2_O_2_, UCMSCs-exo or UCMSCs-exo/eNOS. Scale bar: 30 μm. **c** Histogram showing the ratio of PI/Calcein fluorescence (red/green) in each group of HUVECs (n = 3, two-tailed Student’s t-test). **d** Histograms showing the ratio of JC-1 polymer/JC-1 monomer (red/green) in each group of HUVECs (n = 4, two-tailed Student’s t-test). **e** Representative images showing 2,7-Dichlorodi -hydrofluorescein diacetate (DCF, green) staining for the detection of ROS production in each group of HUVECs. Scale bar: 50 μm. **f** Representative images showing TUNEL staining of apoptotic HUVECs after H_2_O_2_, UCMSCs-exo, or UCMSCs-exo/eNOS treatment. **g** Quantitative analysis of ROS generation (n = 3, two-tailed Student’s t-test). (h) Quantitative analysis of TUNEL-positive cells (n = 5, two-tailed Student’s t-test). (**i**) (**j**) Expression of inflammatory factors (IL6, IL8, and TNF-α) in HUVECs and fibroblasts after H_2_O_2_, UCMSCs-exo, or UCMSCs-exo/eNOS treatment (n = 6, two-tailed Student’s t-test). **k**, **l** Expression of angiogenic and antioxidant-related genes (n = 6, two-tailed Student’s t-test). **m** Expression of genes associated with fibroblast proliferation (n = 6, two-tailed Student’s t-test). Data represent means ± SD. *p < 0.05, **p < 0.01, ***p < 0.001 vs. H_2_O_2_ group; #*p* < 0.05, ##*p* < 0.01, ###*p* < 0.001 vs. UCMSCs-exo group

The effects of UCMSCs-exo/eNOS on damage caused by oxidative stress as a driver of chronic wound healing were then investigated. First, the ability of vascular endothelial cells to resist apoptosis induced by hydrogen peroxide was evaluated. The results showed that UCMSCs-exo/eNOS treatment significantly reduced the number of PI-labeled dead cells while the percentage of live cells stained with Calcein increased significantly (Fig. 4a, c). JC-1, a mitochondria-specific fluorescent dye, was used to explore the effect of UCMSCs-exo/eNOS on hydrogen peroxide-induced changes in the mitochondrial membrane potentials in HUVECs. At higher mitochondrial membrane potentials, JC-1 forms aggregates (J-aggregates) in the mitochondrial matrix, which produce red fluorescence while at lower potentials, JC-1 dissociates into monomers in the mitochondrial matrix, emitting green fluorescence. The results showed a significant increase in the JC-1 polarization ratio that reflected the recovery of the mitochondrial membrane potential after treatment with UCMSCs-exo/eNOS (Fig. 4b, d). These results suggest that treatment with UCMSCs-exo/eNOS ameliorates oxidative stress-induced changes in the mitochondria of vascular endothelial cells. Furthermore, the production of intracellular ROS was evaluated by detecting the fluorescence intensity of DCF. The results showed that UCMSCs-exo/eNOS significantly reduced ROS production in endothelial cells with oxidative stress caused by hydrogen peroxide (Fig. 4e, g). The TUNEL apoptosis assay further demonstrated that UCMSCs-exo/eNOS could counteract hydrogen peroxide-induced endothelial cell apoptosis (Fig. 4f, h).

Analysis of the mRNA expression of inflammatory factors showed that UCMSCs-exo/eNOS reduced the expression of IL6, IL8, and TNF-α and alleviated inflammation more strongly than UCMSCs-exo (Fig. 4i, g). To further verify the biological activity of UCMSCs-exo/eNOS, downstream targets were investigated in endothelial cells and fibroblasts. Compared to the H_2_O_2_ group, UCMSCs-exo/eNOS treatment reversed and upregulated the levels of SIRT1, Nrf2, GPX4, NQO1, and SOD1, indicating resistance against oxidative stress induced by hydrogen peroxide. Meanwhile, increased expression of TGF-β, HO-1, PPARα, VEGFA, CCL2, CXCL1, and PDGFRE demonstrated that UCMSCs-exo/eNOS had the ability to resist endothelial cell injury, promote angiogenic activity, and regulate regeneration-related gene expression (Fig. 4k, i). RAS, TAK1, COL1A1, COL3A1, PDGFa, and PDGFb expression was upregulated in fibroblasts (Fig. 4m), indicating that UCMSCs-exo/eNOS promoted the expression of genes related to the proliferation and migration of fibroblasts and contributed to fibroblast activation.

### UCMSCs-exo/eNOS promote wound closure and stromal remodeling in diabetic mice

**Fig. 5:**
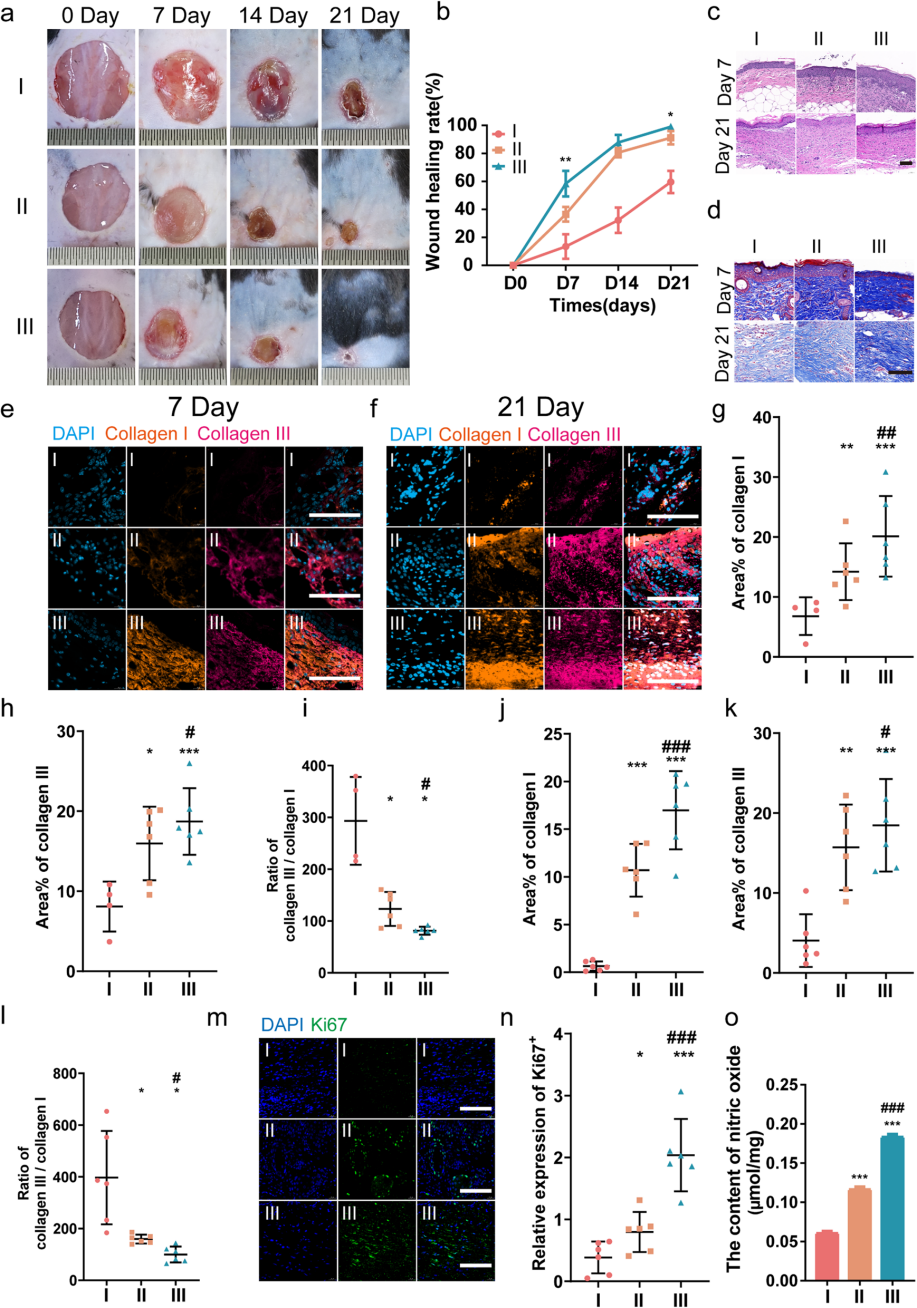
UCMSCs-exo/eNOS promote matrix remodeling and tissue repair in diabetic mouse wounds. I: Treatment with PBS. II: Treatment with UCMSCs-exo. III: Treatment with UCMSCs-exo/eNOS. **a**, **b** Representative images of full-thickness skin defects and wound-healing rates in diabetic mice receiving multi-point injections of PBS, UCMSCs-exo, and UCMSCs-exo/eNOS at postoperative days 0, 7, 14, and 21 (n = 4 in each group at each time point, two-tailed Student’s t-test). **c** H&E staining of wound sections treated with PBS, UCMSCs-exo, and UCMSCs-exo/eNOS at postoperative days 7 and 21 days. **d** Masson staining of wound sections treated with PBS, UCMSCs-exo, and UCMSCs-exo/eNOS at postoperative days 7 and 21. **e** Immunofluorescence staining of Collagen I and Collagen III in the wounds of the different groups on postoperative day 7. Scale bar: 100 μm. **f** Immunofluorescence staining of Collagen I and Collagen III in the wounds of different groups on postoperative day 21. Scale bar: 100 μm. **g**, **h**, **i** Immunofluorescence quantification of Collagen I and Collagen III and the Collagen III to Collagen I ratio in **e** (n = 4 in I group, n = 6 in II group and n = 6 in III group, Mann–Whitney U test). **j**, **k**, **l** Immunofluorescence quantification of Collagen I and Collagen III and the Collagen III to Collagen I ratio in **f** (n = 6 in each group, two-tailed Student’s t-test). **m** Immunofluorescence staining of Ki67 in the wounds of the different groups on postoperative day 7. Scale bar: 100 μm. **n** Immunofluorescence quantification of Ki67 (n = 6 in each group, two-tailed Student’s t-test). **o** Subcutaneous injection of PBS, UCMSCs-exo, and UCMSCs-exo/eNOS into diabetic mice with chronic wounds and analysis of the nitric oxide content at the wound site (n = 3 in each group, two-tailed Student’s t-test). Data represent means ± SD. *p < 0.05, **p < 0.01, ***p < 0.001 vs. PBS group; ^#^p < 0.05, ^##^p < 0.01, ^###^p < 0.001 vs. UCMSCs-exo group

We made full-thickness skin wounds of 1.5 cm in diameter on the dorsal surfaces of db/db mice and treated them with multi-point subcutaneous injections of PBS, UCMSCs-exo, and UCMSCs-exo/eNOS around the wound. The wounds were monitored to study the therapeutic effects of UCMSCs-exo/eNOS on diabetic wound healing (Fig. 5a). Images of the skin defects showed accelerated wound closure over time in the UCMSCs-exo group compared to the PBS group while UCMSCs-exo/eNOS showed enhanced promotion of wound healing at days 7, 14, and 21. Only the UCMSCs-exo/eNOS-treated wounds were almost closed by day 21 (Fig. 5b). No erythema, puffiness, or irritation was observed in the wound area in all three groups during the entire treatment period. We further analyzed histological changes in the wound tissue on days 7 and 21 of treatment. HE-stained sections showed that the diabetic wounds in the UCMSCs-exo/eNOS group exhibited better re-epithelialization, with significantly enhanced granulation tissue formation, more epithelial structures, and longer neuroepithelia (Fig. 5c).

Appropriate collagen deposition and remodeling can improve the biomechanical properties of tissue and promote better healing outcomes. Masson staining showed the presence of thicker large wavy collagen fibers and more extensive collagen deposition in UCMSCs-exo/eNOS-treated skin (Fig. 5d), indicating the superior ability of the UCMSCs-exo/eNOS to promote extracellular matrix remodeling and epithelial regeneration. Types I and III collagen, as major components of the dermal extracellular matrix, play important roles in the wound healing process. In terms of differences in the collagen contents of the three treatment groups, we further analyzed the relationship between UCMSCs-exo/eNOS and the expression levels of collagen I/III in the wound tissue using an immunofluorescence method. Similar to the results of the Masson staining, it was found that the deposition of collagen types I and III was also increased in all wounds throughout the healing phase. However, UCMSCs-exo/eNOS-treated wound tissues had higher densities of types I and III collagen at days 7 (Fig. 5e, g–i) and 21 (Fig. 5f, j–l) of monitoring. Moreover, the most suitable and balanced COL3A1/COL1A1 ratio was observed in the UCMSCs-exo/eNOS group, and greater type III collagen deposition also prevented skin scarring. Combined with the pathological findings of the wounded skin and collagen remodeling promoted by UCMSCs-exo/eNOS, UCMSCs-exo/eNOS led to better healing effects and effectively promoted favorable collagen production.

Ki67 staining of skin tissues was performed at the midpoint of the treatment process to assess the proliferative activity of skin cells at the wound site. Although both UCMSCs-exo and UCMSCs-exo/eNOS groups showed visible Ki67-positive staining, the UCMSCs-exo/eNOS-treated skin showed greater Ki67 expression and enhanced proliferation of Ki67-positive skin cells (Fig. 5m, n).

At the time of wound formation, NO acts to promote vasodilation and inhibit platelet aggregation, as well as defend against aggressive pathogens. At the same time, NO can promote angiogenesis and collagen deposition by stimulating secretion by endothelial cells and fibroblasts [33, 34]. However, decreased NO production in wound tissue can also disrupt the immune state [12]. NO plays an important role throughout the wound-healing process. As eNOS is one of the key enzymes involved in NO production, we examined the effects of exogenous administration of eNOS from UCMSCs-exo/eNOS for NO production in wound tissues. The results showed that UCMSCs-exo/eNOS enhanced NO concentrations at the wound compared to UCMSCs-exo (Fig. 5o). This implies that UCMSCs-exo/eNOS-mediated NO production plays an important role throughout the tissue repair process.

### UCMSCs-exo/eNOS promote angiogenesis in diabetic mouse wounds

**Fig. 6:**
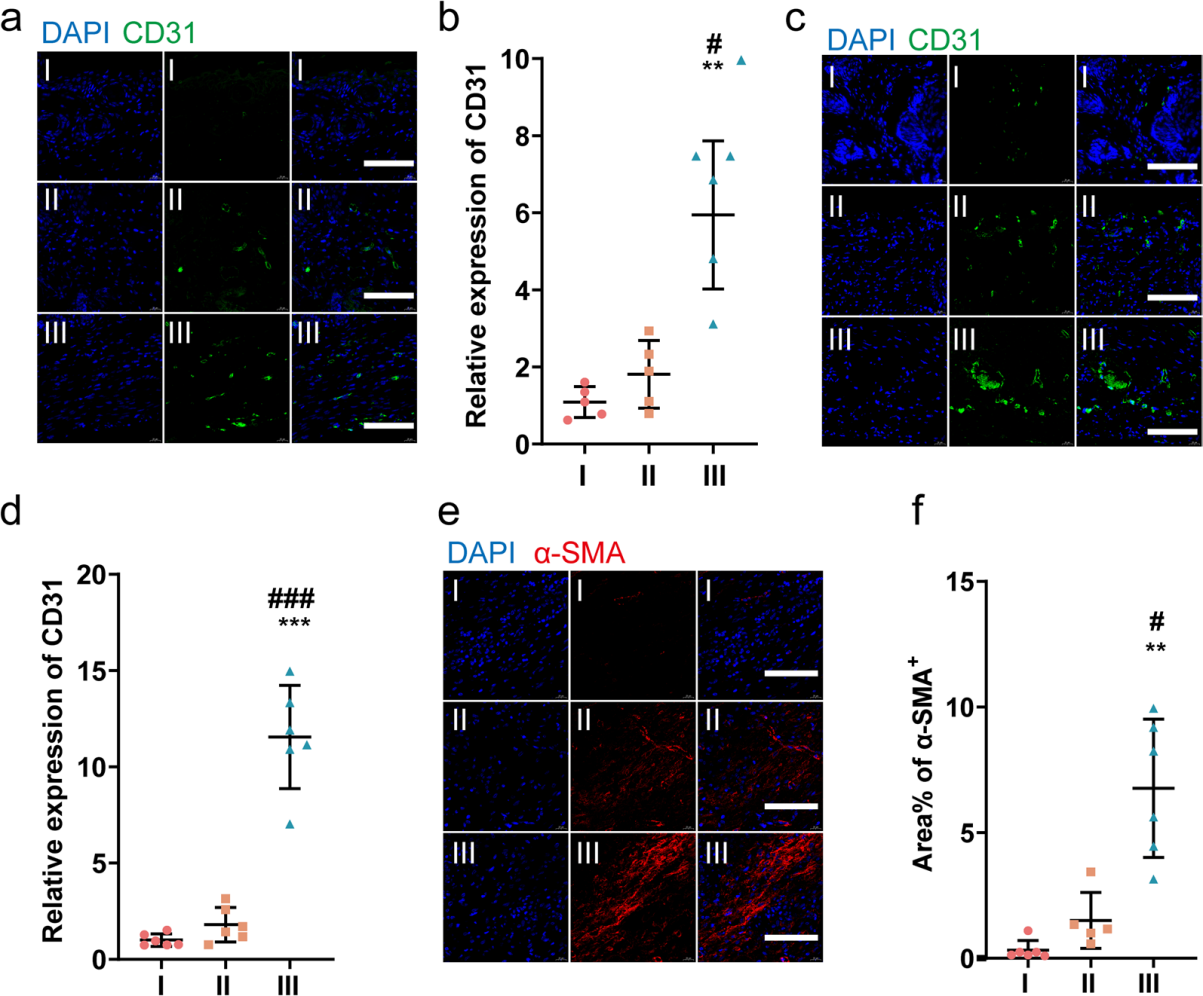
UCMSCs-exo/eNOS promote angiogenesis in chronic wounds of diabetic mice. I: Treatment with PBS. II: Treatment with UCMSCs-exo. III: Treatment with UCMSCs-exo/eNOS. **a** CD31 immunofluorescence staining of neovascularization in the wounds of different groups at postoperative day 7. Scale bar: 100 μm. **b** Quantitative immunofluorescence analysis of CD31 in **a** (n = 6 in each group, two-tailed Student’s t-test). **c** CD31 immunofluorescence staining of neovascularization in the wounds of different groups on postoperative day 21. Scale bar: 100 μm. **d** Quantitative immunofluorescence analysis of CD31 in **c** (n = 6 in each group, two-tailed Student’s t-test). **e** α-SMA immunofluorescence staining of mature blood vessels from the wounds of different groups on postoperative day 21. Scale bar: 100 μm. **f** Quantitative immunofluorescence analysis of α-SMA (n = 6 in I group, n = 5 in II group and n = 6 in III group, Mann–Whitney U test). Data represent means ± SD. *p < 0.05, **p < 0.01, ***p < 0.001 vs. PBS group; ^#^*p* < 0.05, ^##^*p* < 0.01, ^###^*p* < 0.001 vs. UCMSCs-exo group

Because blood vessels deliver oxygen and nutrients to cells in and around the wound area, angiogenesis is considered a key process in tissue healing. Measurement of CD31 expression was used to assess the levels of newly formed blood vessels in the healing tissue. At days 7 (Fig. 6a, b) and 21 (Fig. 6c, d) of treatment, the expression of CD31 was significantly higher in the wounds of UCMSCs-exo/eNOS-treated diabetic mice than in the UCMSCs-exo and PBS groups. The area of neovascularization also increased with time. We assessed the expression of α-smooth muscle actin (α-SMA) at the last monitoring time point to assess the condition of the mature vessels after treatment. Compared to the UCMSCs-exo and PBS groups, the area of α-SMA was significantly higher in UCMSCs-exo/eNOS-treated skin wounds and larger tubular structures could be observed (Fig. 6e, f). These results demonstrate that UCMSCs-exo/eNOS with efficient loading of functional proteins through the optogenetic system show superior prmotion of angiogenesis and vascular maturation in diabetic skin injury.

### UCMSCs-exo/eNOS promote tissue repair of diabetic wounds through multiple phosphorylation cascades and molecular pathways

**Fig. 7:**
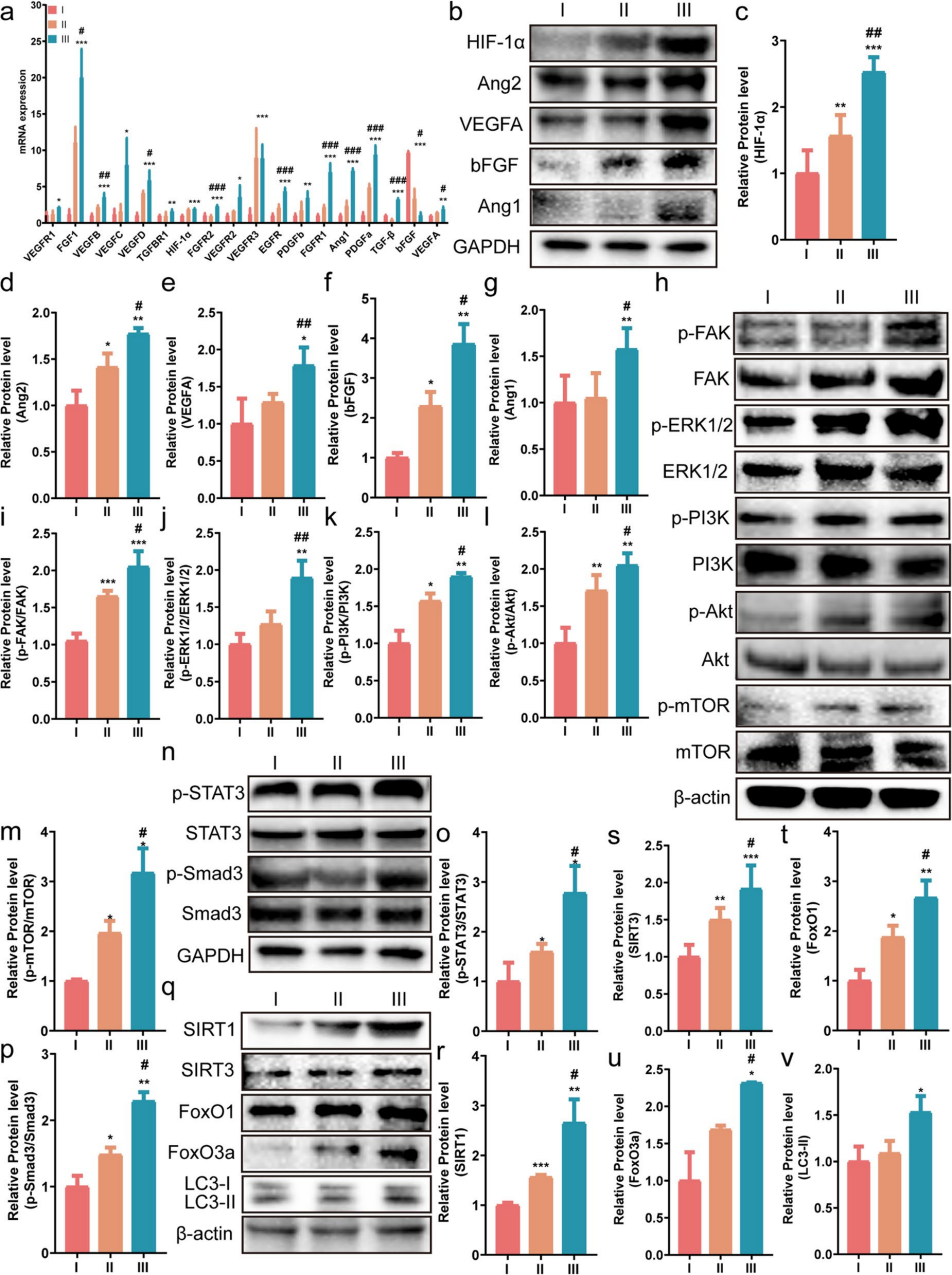
UCMSCs-exo/eNOS promote angiogenesis and chronic wound healing through multiple cascade reactions and molecular activation. I: Treatment with PBS. II: Treatment with UCMSCs-exo. III: Treatment with UCMSCs-exo/eNOS. **a** qRT-PCR analysis of the mRNA expression of angiogenesis-associated and healing-associated factors. **b** Western blot analysis of HIF-1α, Ang2, VEGFA, bFGF, and Ang1 protein expression in the wounds of each group up to day 21 of treatment. **c**, **d**, **e**, **f**, **g** Quantitative analysis of HIF-1α, Ang2, VEGFA, bFGF, and Ang1 expression. **h** Western blot analysis of p-FAK, p-ERK1/2, p-PI3K, p-Akt, and p-mTOR protein expression in wounds of each group at day 21 of treatment. **i**, **j**, **k**, **l**, **m** Quantitative analysis of the phosphorylation of p-FAK, p-ERK1/2, p-PI3K, p-Akt, and p-mTOR. **n** Western blot analysis of p-STAT3 and p-Smad3 expression in the wounds of each group at day 21 of treatment. **o**, **p**, Quantitative analysis of the degree of phosphorylation of p-STAT3 and p-Smad3. **q** Western blot analysis of the protein expression of SIRT3, SIRT1, FoxO1, FoxO3a, and LC3 in the wounds of each group at day 21 of treatment. **r**, **s**, **t**, **u**, **v** Quantitative analysis of SIRT3, SIRT1, FoxO1, FoxO3a, and LC3-II. Results are from at least three independent replicate experiments. Data represent means ± SD. *p < 0.05, **p < 0.01, ***p < 0.001 vs. PBS group; ^#^*p* < 0.05, ^##^*p* < 0.01, ^###^*p* < 0.001 vs. UCMSCs-exo group as determined by two-tailed t-tests

To investigate the potential mechanism by which functionalized UCMSCs-exo/eNOS promote angiogenesis and chronic wound healing, we analyzed the skin lesions of diabetic mice. We first performed gene expression analysis of angiogenesis-related pathways in the skin of mice from different treatment groups and found that UCMSCs-exo/eNOS could promote the expression of genes in some pathways (Additional file 2: Figure S2a-c), which differed from the effects of the UCMSCs-exo. Poor angiogenesis is the main reason for the failure of wound healing in diabetic patients. The RT-qPCR results showed that treatment with UCMSCs-exo/eNOS upregulated the expression of a large number of angiogenic genes in the wound tissue (Fig. 7a). Meanwhile, the western blotting results also showed that UCMSCs-exo/eNOS treatment of mouse skin wounds led to significantly increased expression of the pro-angiogenic factors HIF-1α, Ang1, Ang2, VEGFA, and bFGF, the enhancement of which was stronger than that of UCMSCs-exo (Fig. 7b–g). This may be the main reason why UCMSCs-exo/eNOS are more effective in the promotion of angiogenesis.

However, activation of the PI3K/Akt/mTOR or FAK/ERK1/2 signaling pathways has been shown to enhance skin cell proliferation and migration and can promote angiogenesis. A series of phosphorylation cascade signaling at the wound was found to accelerate the healing of skin wounds. Immunoblotting results showed reduced protein phosphorylation in the PI3K/Akt/mTOR and FAK/ERK1/2 signaling pathways in diabetic mice in the PBS group, while UCMSCs-exo/eNOS best promoted the expression of p-FAK, p-ERK1/2, p-PI3K, p-Akt, and p-mTOR in the new skin tissue, indicating activation of the pathways (Fig. 7h–m). Activation of the PI3K/Akt/mTOR or FAK/ERK1/2 signaling pathways may be one of the potential mechanisms by which UCMSCs-exo/eNOS promote angiogenesis and tissue repair.

Wound healing is a multifactorial cross-linked dynamic process, and we explored other factors involved in the promotion of angiogenesis and wound healing by optogenetically engineered UCMSCs-exo/eNOS. Western blotting showed increased phosphorylation of two proteins, STAT3 and Smad3, after UCMSCs-exo/eNOS intervention (Fig. 7n–p). STAT3 regulates the cellular stress response, apoptosis, and wound-healing pathways [35], while activation of Smad3 promotes tissue repair and reduces local inflammatory cell infiltration [36, 37]. This suggests that UCMSCs-exo/eNOS can regulate tissue repair through multiple pathways in the complex microenvironment of diabetic wounds.

The accumulation of advanced glycosylation end-products (AGEs) is one of the causes of delayed wound healing due to diabetes. However, moderate autophagic activity removes these abnormal proteins or amino acids in vivo and promotes cell repair. To clarify the involvement of autophagic pathways activated by UCMSCs-exo/eNOS in chronic wounds in diabetic mice, we examined the expression of several autophagy-related genes. The results showed that the protein levels of SIRT3, SIRT1, FoxO1, FoxO3a, and LC3-II were significantly upregulated in the skin tissues of mice in the UCMSCs-exo/eNOS group compared to the PBS and UCMSCs-exo groups (Fig. 7q–v). In conjunction with the results of the histology and wound status described above, these data suggest that UCMSCs-exo/eNOS promote diabetic wound repair through appropriate autophagy activation.

### UCMSCs-exo/eNOS reduce inflammatory cell infiltration and the inflammatory response at the wound site

**Fig. 8:**
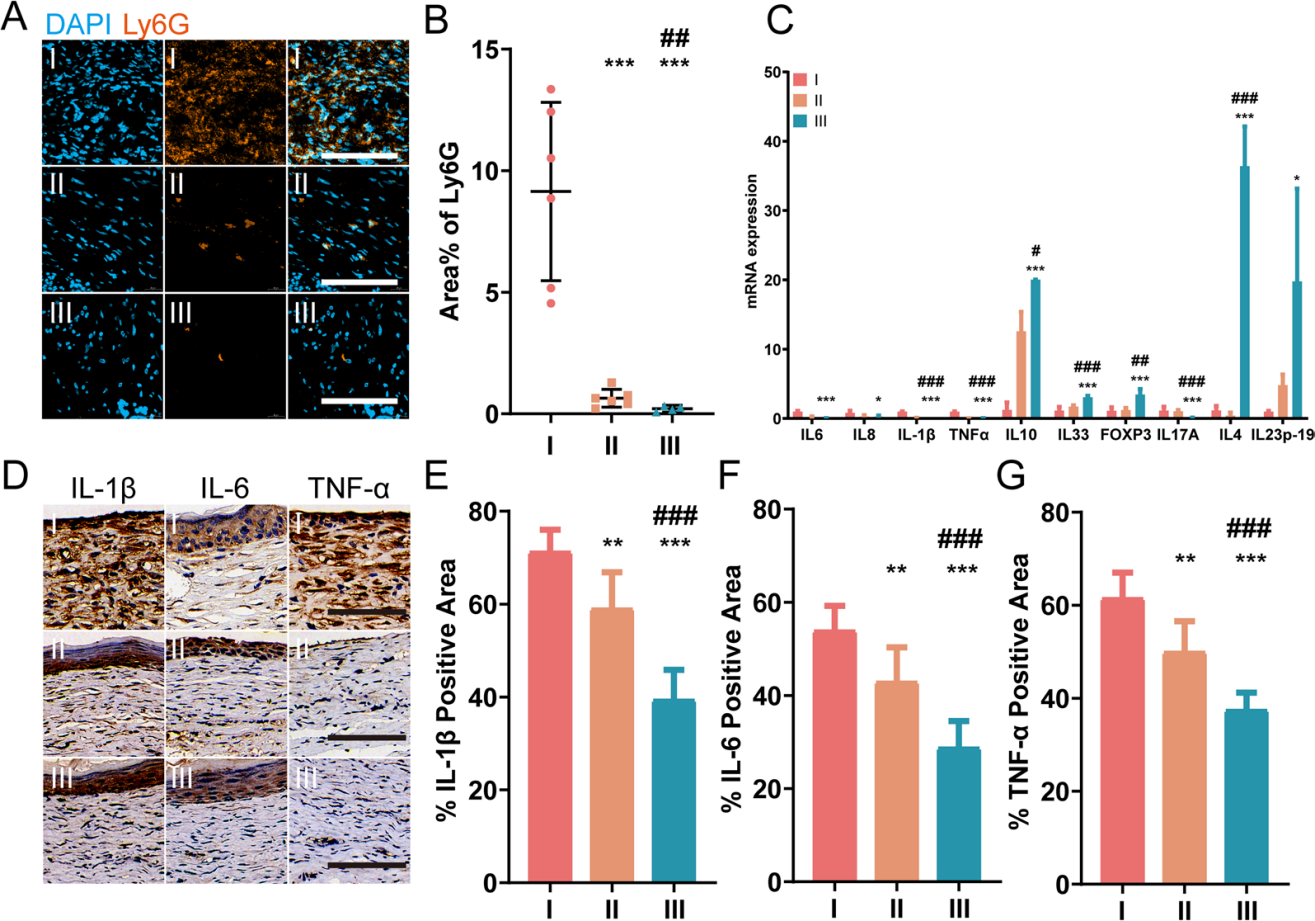
UCMSCs-exo/eNOS inhibit the inflammatory response associated with chronic trauma in diabetic mice. I: Treatment with PBS. II: Treatment with UCMSCs-exo. III: Treatment with UCMSCs-exo/eNOS. (**a**) Immunofluorescence staining of Ly6G in skin wounds of PBS, UCMSCs-exo, and UCMSCs-exo/eNOS-treated diabetic mice on postoperative day 7. Scale bar: 100 μm. (**b**) Immunofluorescence quantification of Ly6G (n = 6 in each group, two-tailed Student’s t-test). (**c**) RT-qPCR analyis of the expression of inflammation-associated cytokines in the wound tissue on treatment day 14 (at least three independent replicate experiments). (**d**) Immunohistochemistry of inflammation-associated cytokines (IL1β, IL6, and TNF-α) in wound tissue on treatment day 14. (**e**) (**f**) (**g**) Quantification of immunohistochemical staining of IL-1β, IL6, and TNF-α (n = 6 in each group, two-tailed Student’s t-test). Data represent means ± SD. *p < 0.05, **p < 0.01, ***p < 0.001 vs. PBS group; #*p* < 0.05, ##*p* < 0.01, ###*p* < 0.001 vs. UCMSCs-exo group

Pathologically persistent and widespread inflammation is one of the main reasons for the disruption of the normal healing cascade response in chronic diabetic wounds. We evaluated neutrophil infiltration at the wound site, observing significant infiltration of Ly6G + cells in the PBS group. In contrast, neutrophil infiltration was significantly reduced in the UCMSCs-exo/eNOS-treated wounds compared to those of the UCMSCs-exo group (Fig. 8a, b). Quantitative RT-PCR results showed that UCMSCs-exo/eNOS were more effective in downregulating the expression of inflammation-related factors. Notably, the Th2 T cell subpopulation-associated cytokines IL10 and IL33 and the Treg-associated cytokine FOXP3 levels were significantly upregulated (Fig. 8c). However, wound healing is a multi-step process and numerous factors are involved in its different stages. Cell–cell and cell–matrix interactions mediated by cell adhesion molecules and chemokines are among the key factors regulating wound healing. We examined the gene expression of adhesion factors and chemokines in skin defects. The results showed that VE-Cadherin expression levels were downregulated in the UCMSCs-exo/eNOS group, but were accompanied by upregulation of ICAM-1, VCAM-1, and PECAM (Additional file 3: Figure S3). However, chemokines can also play a role in preventing or promoting healing at different stages of angiogenesis. The PCR results showed that the expression levels of chemokines CCL2, CXCL1, CXCL5, CCL11, CXCL10, and CXCL11, which are pratly responsible for macrophage recruitment and the direct promotion of angiogenesis and epithelialization, were significantly upregulated in the skin of mice in the UCMSCs-exo/eNOS group, compared with the UCMSCs-exo and PBS groups (Additional file 3: Figure S3). Immunohistochemical analysis of inflammatory cytokines in the skin tissue of each group showed that the positive proportions of IL1β, IL6, and TNF-α were significantly reduced after UCMSCs-exo/eNOS treatment, signifying inhibition of the inflammatory response (Fig. 8d–g).

### UCMSCs-exo/eNOS reshape the immune microenvironment of wound tissue

**Fig. 9:**
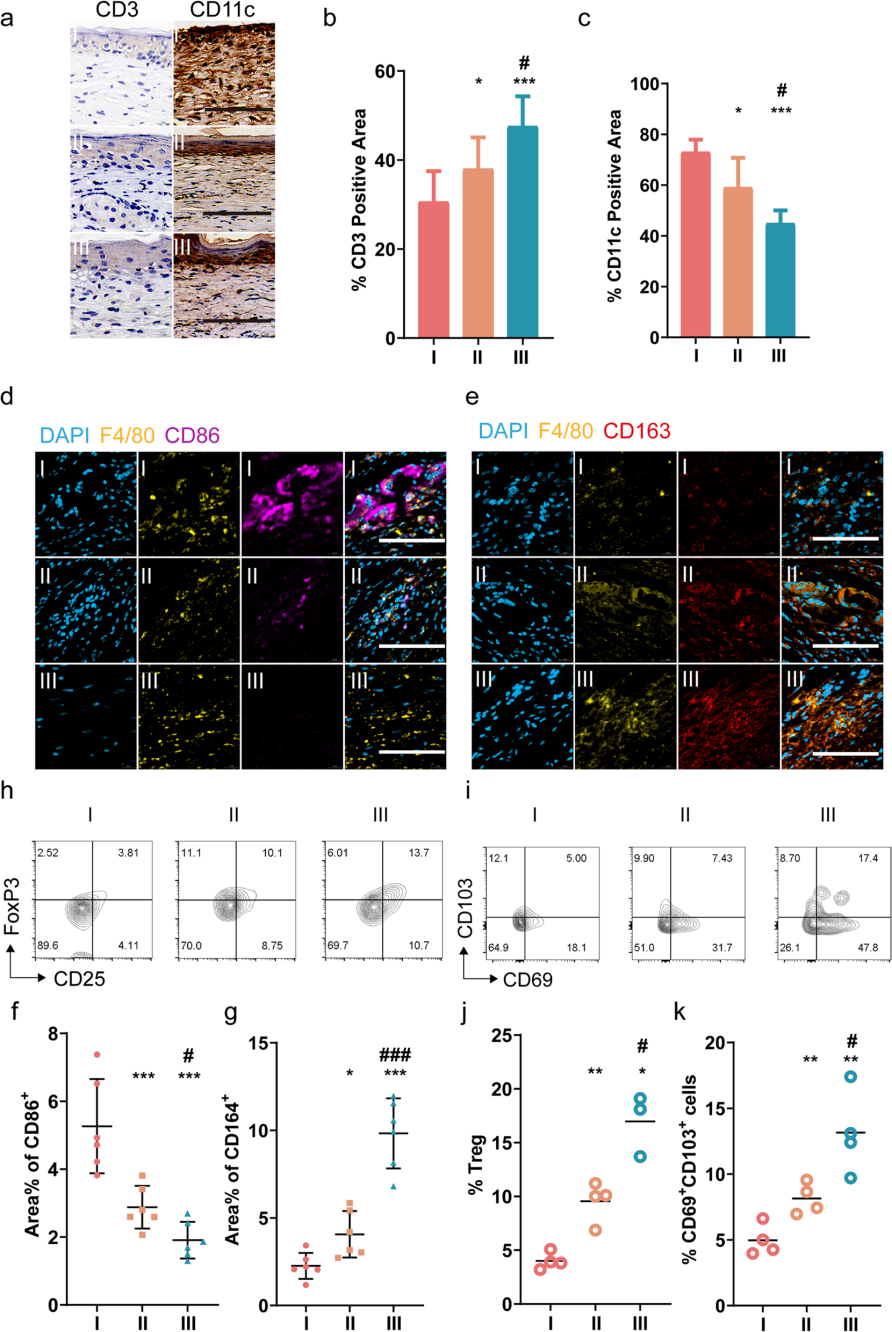
Remodeling of the immune microenvironment of the chronic wounds in diabetic mice by UCMSCs-exo/eNOS. I: Treatment with PBS. II: Treatment with UCMSCs-exo. III: Treatment with UCMSCs-exo/eNOS. **a** Immunohistochemistry of CD3 and CD11c in the skin wounds of diabetic mice treated with PBS, UCMSCs-exo, and UCMSCs-exo/eNOS on postoperative day 14. Scale bar: 100 μm. **b**, **c** Quantification of immunohistochemical staining of CD3 and CD11c (n = 6 in each group, two-tailed Student’s t-test). **d** M1 macrophages in the wound tissue. immunofluorescence images of F4/80 and CD86. Scale bar: 100 μm. **e** M2 macrophages in the wound tissue: immunofluorescence images of F4/80 and CD163. Scale bar: 100 μm. **f**, **g** Quantitative immunofluorescence analysis of M1 and M2 macrophages(n = 6 in each group, two-tailed Student’s t-test). **h** Representative flow cytometry plots of CD25 + Foxp3 + Treg cells in wound tissue. **i** Representative flow cytometry image of CD69 + CD103 + T_RM_ cells in wound tissues. **j** Quantitative analysis of the proportion of CD25 + Foxp3 + Treg cells in wound tissues (n = 4 in I group, n = 4 in II group and n = 3 in III group, Mann–Whitney U test). **k** Quantitative analysis of the proportion of CD69 + CD103 + T_RM_ cells in wound tissues (n = 4 in each group, two-tailed Student’s t-test). Data represent means ± SD. *p < 0.05, **p < 0.01, ***p < 0.001 vs. PBS group; #*p* < 0.05, ##*p* < 0.01, ###*p* < 0.001 vs. UCMSCs-exo group

Immune cells remove tissue debris and microbial contamination in wounded tissue and also secrete cytokines and growth factors that promote tissue repair and wound closure. A discussion of the immune environment in tissues during the treatment phase is necessary. We performed immunohistochemical analysis of the wounded skin tissues and showed that the area of CD3 positivity was highest in the skin of mice in the UCMSCs-exo/eNOS group, suggesting that the percentage of CD3 + T cells in the tissues had increased due to treatment. In contrast, the high expression of CD11c in the PBS group suggested the presence of severe dendritic cell (DC) infiltration in the skin, while treatment with UCMSCs-exo/eNOS alleviated the accumulation of DC cells at the wound site (Fig. 9a–c).

Macrophages promote wound repair and tissue remodeling by reducing the autoimmune response and chronic inflammatory response [38]. However, the hyperglycemic environment leads to polarization of macrophages toward a proinflammatory phenotype, resulting in difficulties in wound healing [22]. To investigate the effect of UCMSCs-exo/eNOS treatment on macrophage polarization, we characterized the phenotype of macrophages by multiple immunofluorescence staining. The results showed the highest percentage of CD86-positive cells and the lowest percentage of CD163-positive cells in the PBS group, which indicated that a large number of M1 pro-inflammatory macrophages were residing in the wound tissue (Fig. 9d, f). In contrast, the skin of UCMSCs-exo/eNOS-treated mice showed reversal of this state, and the wounded tissue expressed a large number of CD163-positive cells, implying that UCMSCs-exo/eNOS could induce the polarization of macrophages at the wound to the M2 anti-inflammatory phenotype (Fig. 9e, g).

Treg cells can promote tissue repair by eliminating tissue inflammation and secreting cytokines and growth factors. By analyzing skin cells from different groups, we found that both UCMSCs-exo and UCMSCs-exo/eNOS increased the proportions of of CD4 + FoxP3 + CD25 + cells, while UCMSCs-exo/eNOS significantly enhance Treg presence in the skin (Fig. 9h, j).

Bacterial infection is one of the barriers to diabetic wound healing, and slowed closure and open wounds allow increased exposure to and invasion of pathogens. Tissue-resident memory CD8 + T cells (T_RM_) cells protect and maintain the integrity of the barrier surface against secondary exposure to various pathogens. We analyzed alterations in the T_RM_ numbers in each group during the healing process, with the flow cytometry results showing that both UCMSCs-exo and UCMSCs-exo/eNOS treatment increased the numbers of CD8 + CD69 + CD103 + T_RM_ cells in the skin tissue, while UCMSCs-exo/eNOS treatment resulted in significantly greater numbers of CD8 + CD69 + CD103 + T_RM_ cells present in the skin tissue around the wound (Fig. 9i, k). These results suggest that UCMSCs-exo/eNOS create a regenerative immune microenvironment for tissue repair by regulating the aggregation of Treg cells in injured tissues, and promoting the residence of CD8 + CD69 + CD103 + T_RM_ cells to provide surveillance against pathogens and protection of the organism.

## Discussion

The hyperglycemic state interferes with the various stages of wound healing in diabetic patients, and acute skin wounds slowly develop into chronic non-healing wounds as the repair process cannot be concluded. Various therapeutic strategies have been attempted to resolve the problem of the healing of chronic wounds, but the optimal treatment strategies still require development. We enabled efficient and massive loading of the exogenous therapeutic protein eNOS into UCMSC-derived exosomes to enhance their pro-angiogenic and tissue repair abilities through optogenetic-based protein–protein module interaction techniques. Furthermore, we evaluated their effects on chronic non-healing wounds in diabetic mice and demonstrated that UCMSCs-exo/eNOS exerted superior therapeutic effects over MSC-derived exosomes in diabetic wound repair by suppressing the inflammatory state, promoting angiogenesis, improving the microenvironment for tissue remodeling, and modulating the immune response around the wound. This study presents a novel optogenetically engineered stem cell-derived exosome treatment that optimizes the enrichment of exogenous functional proteins in UCMSC-derived exosomes and provides a more effective cell-free therapeutic strategy for angiogenesis and tissue repair in chronic diabetic wounds.

MSCs, as pluripotent cells with differentiation and immunomodulatory properties, have significant tissue regenerative capacity [39]. MSCs secrete vascular growth factors that promote tissue repair by enhancing angiogenesis during wound healing. In contrast, UCMSCs are easily available in large quantities and have lower immunogenicity, greater potential for differentiation and tissue repair, and are more likely to survive in the culture conditions of the skin [40, 41]. This led us to select UCMSCs as the basis for genetic and optogenetic engineering to perpetuate their excellent targeting of injury sites and tissue repair functions.

MSCs affect neighboring cell biological functions through paracrine signaling, including exosome secretion [42, 43]. MSCs-exo-based therapy is an attractive strategy for tissue repair engineering, a cell-free therapy that avoids immune rejection and ectopic tissue formation [9]. We genetically engineered the therapeutic protein-eNOS to be actively loaded into UCMSC-exo by an endogenous biogenesis process for controlled delivery to target tissues. eNOS is one of three enzymes that promote the conversion of L-arginine to NO. Nitric oxide activity is essential for wound collagen accumulation and the acquisition of mechanical strength [12], are essential for wound healing. The amount of NO is also dependent on the synthesis of eNOS, which is structurally expressed by endothelial cells [44]. NO has been shown to have functions in promoting angiogenesis, proliferation, and migration of both endothelial and epithelial cells [45]. We also detected increased NO contents in the healing wounds of diabetic mice treated with UCMSCs-exo/eNOS, which may account for the more effective pro-angiogenic, tissue remodeling, and immune microenvironmental effects of functionalized UCMSCs-exo/eNOS when combined with the powerful tissue repair capacity of stem cell exosomes.

The mechanism by which the high-glucose environment impedes angiogenesis is a key reason for the difficulty of diabetic wound healing [46]. Diabetic vascular complications with endothelial cell dysfunction ultimately lead to impaired repair and abnormal angiogenesis [47]. Furthermore, hyperglycemia-induced endothelial cell hypofunction is an important cause of vascular malfunction [20]. We observed that UCMSCs-exo/eNOS significantly reversed and improved the biological function of vascular endothelial cells damaged by high glucose and promoted HUVEC proliferation, migration, and tube formation. Under UCMSCs-exo/eNOS intervention, the expression of concomitant inflammatory factors was significantly reduced, ROS production and lipid peroxidation induced by high glucose were inhibited, and the antioxidant capacity of endothelial cells was enhanced, thus controbuting to the maintenance of the redox balance. At the in vivo level, we found that UCMSCs-exo/eNOS had a significant pro-angiogenic and pro-vascular maturation capacity stronger than stem cell-derived exosomes, shown by fluorescent labeling of CD31 and α-SMA. Together with accelerated collagen deposition, UCMSCs-exo/eNOS effectively enabled the repair of chronic wounds. In addition to causing oxidative stress damage to vascular endothelial cells [48], high glucose can also induce endothelial cell apoptosis via signaling [49]. Based on fluorescence imaging of individual assay probes, we demonstrated that UCMSCs-exo/eNOS enhanced endothelial cell resistance to oxidative stress and minimized the levels of apoptosis. Moreover, the expression of individual antioxidant-related genes such as SIRT1, Nrf2, GPX4, and NQO1 was significantly upregulated after UCMSCs-exo/eNOS intervention, which may be one of the reasons for their assistance in protecting endothelial cells from oxidative damage. In addition, upregulation of the expression of several angiogenesis-related genes was observed and the effect was superior to the treatment with common stem cell-derived exosomes. Dysfunctional fibroblast differentiation, disrupted myofibroblast activity, and insufficient extracellular interstitial secretion interfere with wound healing in diabetic patients [50]. Oxidative stress caused by high-glucose conditions in diabetic patients ultimately affects fibroblast malfunction [51]. Whereas persistent inflammation and retarded fibroblast differentiation are also present at the wound site, delayed wound closure is accompanied by reduced epithelial and connective tissue remodeling [52]. We found that UCMSCs-exo/eNOS enhanced the biological functions of high glucose-suppressed fibroblasts, suppressed inflammatory factor alterations brought about by oxidative stress, and upregulated proliferation-related gene expression. Interestingly, at the in vitro level, neither UCMSCs-exo nor UCMSCs-exo/eNOS reversed the expression of COL3A1 and COL1A1 that were inhibited by H_2_O_2_ in fibroblasts. In contrast, the q-PCR analysis of skin tissues from diabetic mice showed different results, indicating upregulation of COL3A1 and COL1A1 expression in the skin accompanied by upregulated expression of genes associated with the degradation and remodeling of the extracellular matrix, as well as upregulation of genes linked to fibroblast proliferation (Additional file 2: Figure S2–S3). We speculate that this may be a result of fibroblast heterogeneity [50], and that different subpopulations of fibroblasts in diabetic wounds may perform specific functions, which is difficult to analyze using a single cell line in in vitro experiments. The biological response of vascular superoxide in vivo has been found to reduce NO and endothelial-type eNOS activity [44]. Reduced NO contents in diabetic trauma have also been shown to be associated with significantly lower eNOS protein expression [13, 14]. Mice lacking the eNOS gene exhibit delayed wound closure and and impaired capillary growth [15]. We enabled the delivery of exogenous eNOS in a biologically spontaneous form to the injury site and optimized a cell-free therapy strategy based on mesenchymal stem cells.

A variety of molecular mechanisms and signaling pathways are involved in the different stages of diabetic wound healing. We investigated the molecular mechanisms by which UCMSCs-exo/eNOS exert their role in promoting angiogenesis and tissue repair. Increased levels of protein phosphorylation marked the activation of the PI3K/Akt/mTOR or FAK/ERK1/2 pathways and, compared with the degree of activation of pathway signaling by UCMSCs-exo, UCMSCs-exo/eNOS were more effective. Together with accelerated angiogenesis, collagen deposition, and the transformation of the microenvironment at the wound, signaling by the PI3K/Akt/mTOR and FAK/ERK1/2 pathways explains the possible potential cause. Disruption of the PI3K/AKT signaling pathway is involved in the development of diabetic ulcers [18]. Activation of AKT/mTOR regulates the expression of growth factors such as VEGF, bFGF, and EGF, promoting cell growth and migration, angiogenesis, and collagen synthesis [53]. Downregulation of ERK1/2 inhibits the proliferation, migration, and collagen expression of fibroblasts [54]. Moreover, the increased phosphorylation levels of FAK and ERK1/2 promote the proliferation and migration of keratinized cells and accelerate the healing of skin wounds [55]. In addition, we found that UCMSCs-exo/eNOS induced enhanced phosphorylation of STAT3 and Smad3 in the wound, suggesting that in addition to activation of the classical cascade pathway, activation of other molecules may be involved in wound angiogenesis and tissue repair. STAT3 regulates the cellular stress response, as well as apoptosis and wound-healing pathways [35] while Smad3 induces the expression of alpha-SMA, VEGF, and TGF-β1 in dermal fibroblasts and induces an increased chemotactic response, accelerating tissue repair in skin ulcers. [36]

Autophagy plays an important role in all stages of wound healing. Autophagy-related gene expression in wound tissue of untreated diabetic mice was at low levels. Notably, the intervention of UCMSCs-exo/eNOS resulted in significant upregulation of SIRT1, SIRT3, FoxO1, FoxO3a, and LC3-II expression in skin tissues. This suggests that either UCMSCs-exo or UCMSCs-exo/eNOS can regulate the level of autophagy at the wound, while engineered UCMSCs-exo/eNOS show superior promotion of autophagy. During the inflammatory phase, autophagy can exert anti-infective effects and negatively regulate the inflammatory response, preventing excessive inflammatory responses leading to tissue damage [56]. During the remodeling phase, autophagy provides resistance against oxidative stress thus promoting cell survival and angiogenesis at the wound site [56]. FOXO3a improves the function of endothelial progenitor cells (EPCs) through autophagy [57] and the activation of the SIRT3 pathway improves the inflammatory condition of the skin [58]. SIRT1 activation also upregulated the levels of VEGF, CD31, and α-SMA, facilitating wound healing [59]. It is worth mentioning that autophagy can enhance macrophage polarization toward the M2 phenotype to reduce the inflammatory response and promote tissue repair [60, 61]. This was demonstrated by our fluorescent labeling of macrophages at the wound site. We explored possible mechanisms related to UCMSCs-exo/eNOS play in chronic wound healing, and the activation of multiple molecular activities and pathways demonstrated the complexity of the various stages of wound healing. These molecules function either synergistically or in parallel further promote angiogenesis and tissue remodeling.

UCMSCs-exo/eNOS enriched with therapeutic proteins were found to remodel the local microenvironment of chronic wounds in diabetic mice by activating downstream pathways and producing NO, together with enhancing the ability of UCMSCs-exo to restore dynamic immune homeostasis. We observed that inflammation levels in diabetic mouse wounds were significantly downregulated by UCMSCs-exo/eNOS treatment, and cytokine expression was also modulated. Thus, UCMSCs-exo/eNOS promoted immune homeostasis in a mouse diabetic wound model. We demonstrated that UCMSCs-exo/eNOS treatment remodeled the immune microenvironment at the wound site by modulating neutrophil infiltration and the anti-inflammatory phenotype of macrophages, as well as the recruitment of Treg cells, suggesting that UCMSCs-exo/eNOS enhance the ability of UCMSCs-exo to regulate the local microenvironment of the defective skin. More importantly, exosomes secreted by MSCs could inhibit M1 polarization and promote M2 polarization to reduce the inflammatory response, and enhance the rate of wound healing by regulating M2 polarization [62]. In contrast, UCMSCs-exo/eNOS showed a superior ability to increase the proportions of Treg and T_RM_ cells in and around the skin defect at the wound site. Given the strong ability of Tregs to control the inflammatory response [63] and the efficient immunosurveillance properties of T_RM_ cells [64], UCMSCs-exo/eNOS showed a stronger ability to remodel the immune environment at the injury site due to their pre-existing anti-inflammatory ability and immunomodulatory potential.

## Conclusions

In conclusion, the application of optogenetic and genetic engineering of UCMSCs-exo/eNOS can accelerate the healing of chronic diabetic wounds by promoting angiogenesis and remodeling the immune microenvironment due to the efficient enrichment of eNOS and the excellent tissue repair and immunomodulatory ability of UCMSCs-exo. In this study, UCMSCs-exo/eNOS enhanced resistance to damage caused by high glucose and immunomodulation at the wound site. In addition, UCMSCs-exo/eNOS enhanced the therapeutic effects of UCMSCs-exo on diabetic wounds. Moreover, we elucidated the molecular mechanism by which UCMSCs-exo/eNOS can promote tissue repair through the activation of phosphorylation cascades and induction of autophagy. In addition, we demonstrated that UCMSCs-exo/eNOS could effectively remodel the local microenvironment and modulate the immune response within wounds. The development of this engineered exosome provides a more effective strategy for MSC exosome-based tissue repair treatment of chronic diabetic wounds.

## Supplementary Information

**Additional file 1: Figure S1.** UCMSCs-exo/eNOS restores inhibition and oxidative damage to the biological functions of fibroblasts and vascular endothelial cells by high glucose.

**Additional file 2: Figure S2.** RT-qPCR results showed the expression of genes in angiogenesis-related pathways.

**Additional file 3: Figure S3.** RT-qPCR showing the expression of adhesion factors and chemokines in traumatic tissues up to day 14 after treatment.

**Additional file 4: Table S1.** Primer sequences.

## Data Availability

The data sets, analytical procedures and experimental details used in the current study are available from the corresponding author upon reasonable request.
