# Supplementary figures and images for: Optogenetic engineered umbilical cord MSC-derived exosomes for remodeling of the immune microenvironment in diabetic wounds and the promotion of tissue repair
Source: J Nanobiotechnology. 2023 Jun 2;21:176. doi: 10.1186/s12951-023-01886-3 (PMC10236791; doi:10.1186/s12951-023-01886-3)

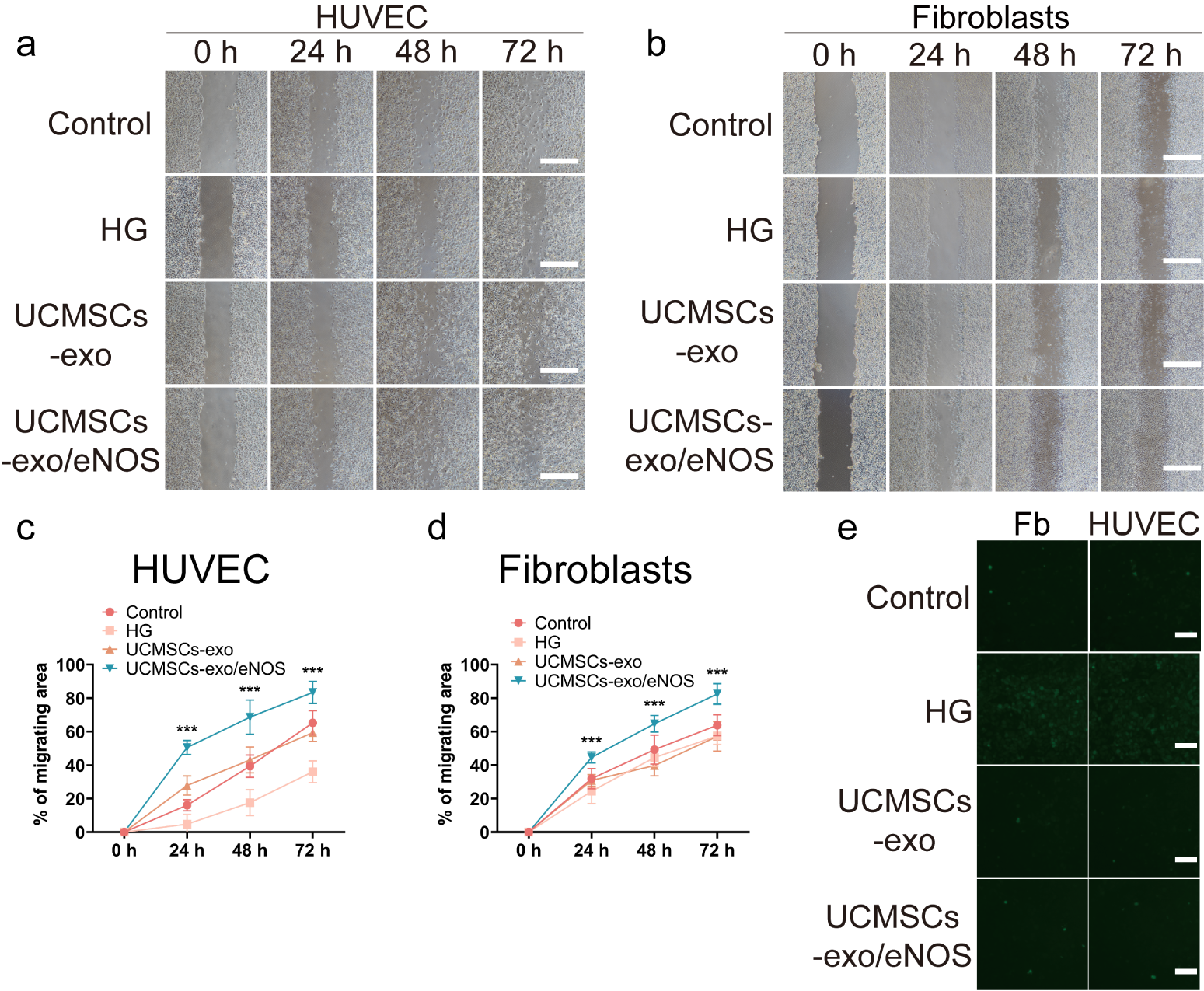

Supplement: Supplementary file 1 — Additional file 1: Figure S1. UCMSCs-exo/eNOS restores inhibition and oxidative damage to the biological functions of fibroblasts and vascular endothelial cells by high glucose. [file 12951_2023_1886_MOESM1_ESM.tif]

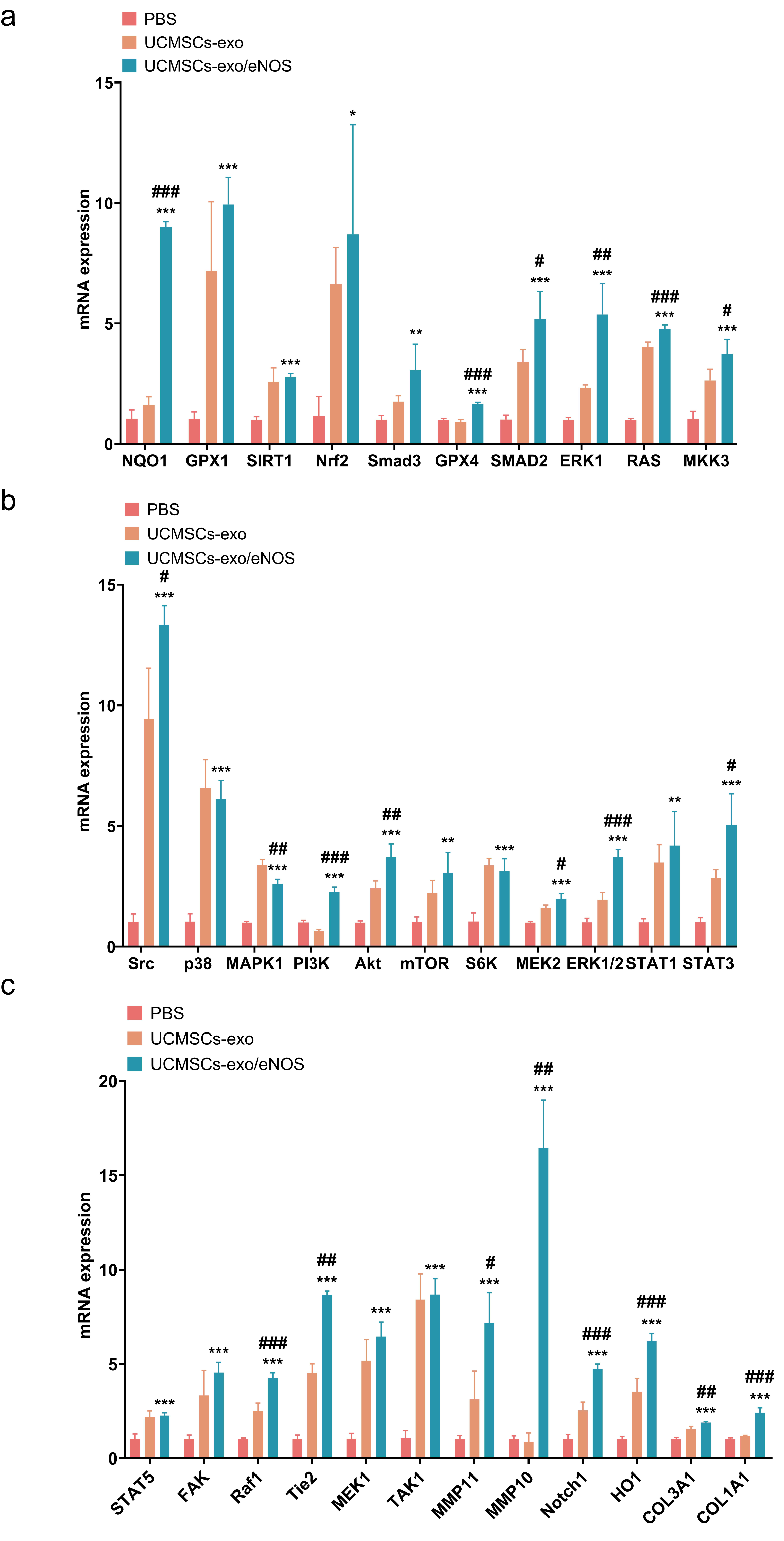

Supplement: Supplementary file 2 — Additional file 2: Figure S2. RT-qPCR results showed the expression of genes in angiogenesis-related pathways. [file 12951_2023_1886_MOESM2_ESM.tif]

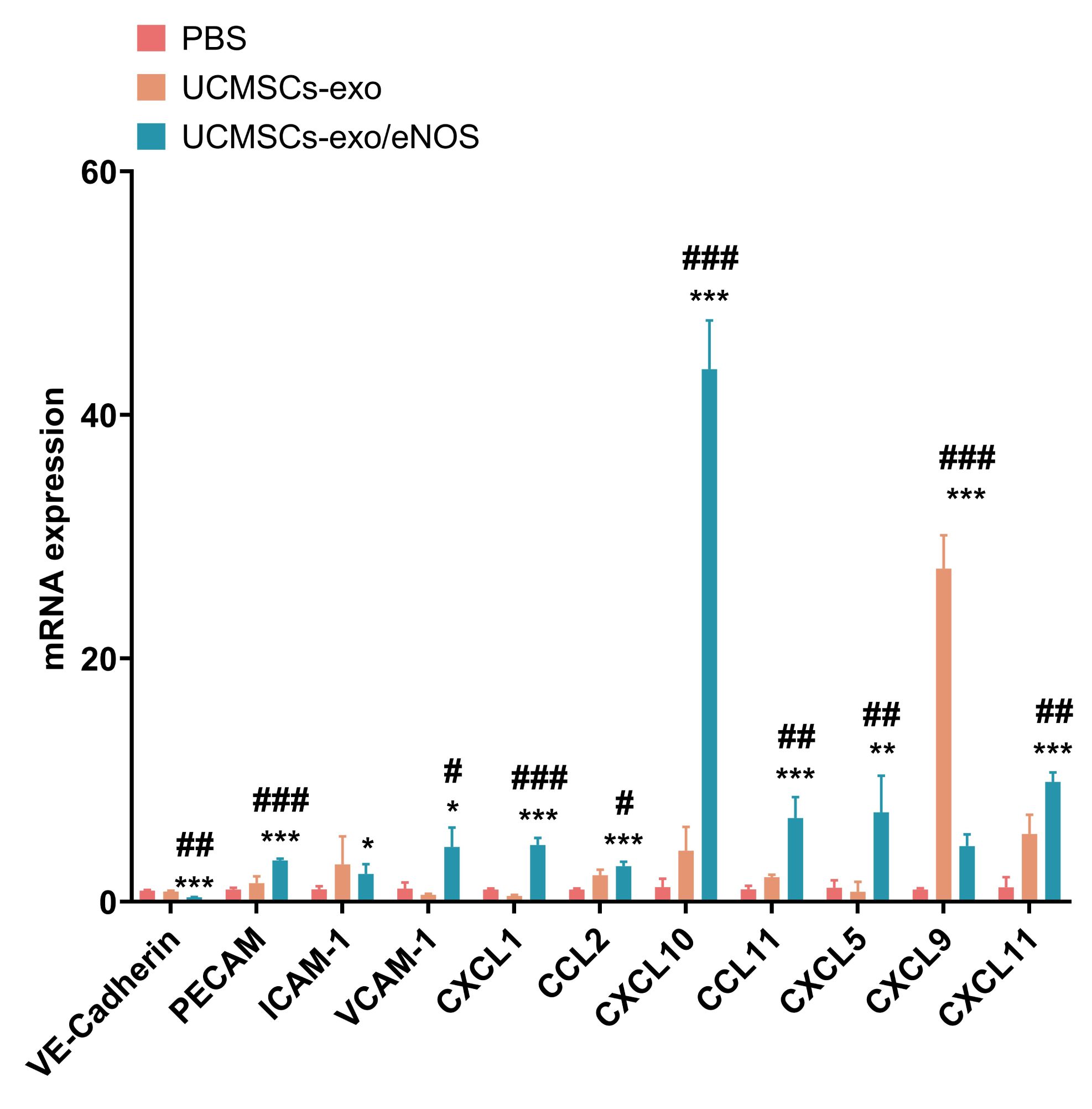

Supplement: Supplementary file 3 — Additional file 3: Figure S3. RT-qPCR showing the expression of adhesion factors and chemokines in traumatic tissues up to day 14 after treatment. [file 12951_2023_1886_MOESM3_ESM.tif]
